# Mechanism-based targeting of cardiac arrhythmias by phytochemicals and medicinal herbs: A comprehensive review of preclinical and clinical evidence

**DOI:** 10.3389/fcvm.2022.990063

**Published:** 2022-09-29

**Authors:** Danesh Soltani, Bayan Azizi, Roja Rahimi, Azita H. Talasaz, Hossein Rezaeizadeh, Ali Vasheghani-Farahani

**Affiliations:** ^1^Cardiac Primary Prevention Research Center (CPPRC), Cardiovascular Diseases Research Institute, Tehran University of Medical Sciences, Tehran, Iran; ^2^Tehran Heart Center, Cardiovascular Diseases Research Institute, Tehran University of Medical Sciences, Tehran, Iran; ^3^Department of Traditional Pharmacy, School of Persian Medicine, Tehran University of Medical Sciences, Tehran, Iran; ^4^Evidence-Based Evaluation of Cost-Effectiveness and Clinical Outcomes, The Institute of Pharmaceutical Sciences (TIPS), Tehran University of Medical Sciences, Tehran, Iran; ^5^Department of Pharmacotherapy and Outcomes Science, Virginia Commonwealth University, Richmond, VA, United States; ^6^Department of Persian Medicine, School of Traditional Medicine, Tehran University of Medical Sciences, Tehran, Iran

**Keywords:** cardiac arrhythmias, antiarrhythmic agents, plant extracts, phytochemicals, herbal medicine

## Abstract

Cardiac arrhythmias, characterized by an irregular heartbeat, are associated with high mortality and morbidity. Because of the narrow therapeutic window of antiarrhythmic drugs (AADs), the management of arrhythmia is still challenging. Therefore, searching for new safe, and effective therapeutic options is unavoidable. In this study, the antiarrhythmic effects of medicinal plants and their active constituents were systematically reviewed to introduce some possible candidates for mechanism-based targeting of cardiac arrhythmias. PubMed, Embase, and Cochrane library were searched from inception to June 2021 to find the plant extracts, phytochemicals, and multi-component herbal preparations with antiarrhythmic activities. From 7337 identified results, 57 original studies consisting of 49 preclinical and eight clinical studies were finally included. Three plant extracts, eight multi-component herbal preparations, and 26 phytochemicals were found to have antiarrhythmic effects mostly mediated by affecting K^+^ channels, followed by modulating Ca^2+^ channels, upstream target pathways, Na_*v*_ channels, gap junction channels, and autonomic receptors. The most investigated medicinal plants were *Rhodiola crenulata* and *Vitis vinifera.* Resveratrol, Oxymatrine, and Curcumin were the most studied phytochemicals found to have multiple mechanisms of antiarrhythmic action. This review emphasized the importance of research on the cardioprotective effect of medicinal plants and their bioactive compounds to guide the future development of new AADs. The most prevalent limitation of the studies was their unqualified methodology. Thus, future well-designed experimental and clinical studies are necessary to provide more reliable evidence.

## Introduction

Cardiac arrhythmias are characterized by disturbances in the heart’s electrical activity and contribute greatly to cardiovascular morbidity and mortality (1). Although antiarrhythmic drugs (AADs) are still one of the most important therapeutic options, several challenges in their administration, including their narrow therapeutic window and adverse drug reactions, remain to be solved and should be considered in future drug discovery (2). Therefore, investigating novel, safe, and effective therapeutic options with antiarrhythmic activity is unavoidable.

Several common AADs drugs, including digoxin, lidocaine, atropine, and amiodarone have originated from medicinal plants (3); thus, researchers have been eager to extend their knowledge about the potential benefits and mechanism of actions of plants and their active ingredients. Over the past two decades, several studies have been designed to show the impact of plants or their ingredients on cardiovascular disease (CVDs); however, the current dearth of information about the clinical aspects of plants renders their application limited (4–6).

This study provides an overview of the preclinical and clinical studies investigating the effects of plant extracts, multi-component herbal preparations, and phytochemicals on cardiac arrhythmias. We also practically categorized these plant-derived medicines based on their mechanism of antiarrhythmic action according to the classification of conventional AADs, dividing them into eight drug classes involving: (0) blockers of the hyperpolarization-activated cyclic nucleotide-gated (HCN) channel-mediated current (*I*_*f*_), (I) blockers of the voltage-gated Na^+^ (Na_*v*_) channels, (II) autonomic inhibitors and activators, (III) K^+^ channel blockers and openers, (IV) Ca^2+^ handling modulators, (V) mechanosensitive transient receptor potential channels subfamily C (TRPC3/TRPC6) blockers, (VI) gap junction channel modulators, and (VII) upstream target modulators (7). We believe that this categorization can confer practitioners a better understanding of the potential benefits of medicinal plants and persuade researchers to design randomized clinical trials aimed at clarifying the efficacy of plant extracts and phytochemicals. We also highlighted the research gaps in previous studies and discussed future research directions.

## Materials and methods

A systematic search was performed using the Medline/PubMed, Embase, and Cochrane Library databases from inception to June 2021. Also, the references of the included studies were reviewed to find potentially relevant research. The keywords used were: (“medicinal plants” OR “herbal medicine” OR “phytochemical” OR “plant extract”) AND (“arrhythmia” OR “antiarrhythmic”). Inclusion criteria were any original English-language preclinical study in which the antiarrhythmic effect and mechanism of antiarrhythmic action of a herbal extract, a plant-derived active compound, or a multi-component herbal preparation were evaluated. We also included all clinical trials that assessed the antiarrhythmic effects of herbal medicine. Reviews, case reports, and original studies with insufficient data were excluded. After removing duplicates, the titles and abstracts were evaluated for eligibility by two independent investigators. Selected studies were further assessed based on their full text. Information on the study design, dose range tested, the model used (*in vitro* or *in vivo* study), type of controls (positive or negative), type of extract, active constituent or multi-component herbal preparation, study duration, antiarrhythmic effects, and mechanism of action were extracted from included studies.

The scientific quality of included animal studies was assessed according to the SYRCLE risk of bias (RoB) tool. This tool consists of ten entries with signaling questions to assist the judgment process in assessing selection bias, performance bias, detection bias, attrition bias, reporting bias, and other sources of bias. A “yes” response to each question represents a low risk of bias, which leads the study to high quality. A “No” response to each question shows a high risk of bias and favors the low quality of the study. An unclear risk of bias means that the information is insufficient to make a decision (8). The quality assessment of clinical studies was performed using Jadad scoring system. This scale determines scores from 1 to 7 points and, thereafter, evaluates the clinical studies as follows: high quality (score >4), moderate quality (score of 3 or 4), and low quality (score of <3) (9).

## Results

### The characteristics and quality of selected studies

**FIGURE 1:**
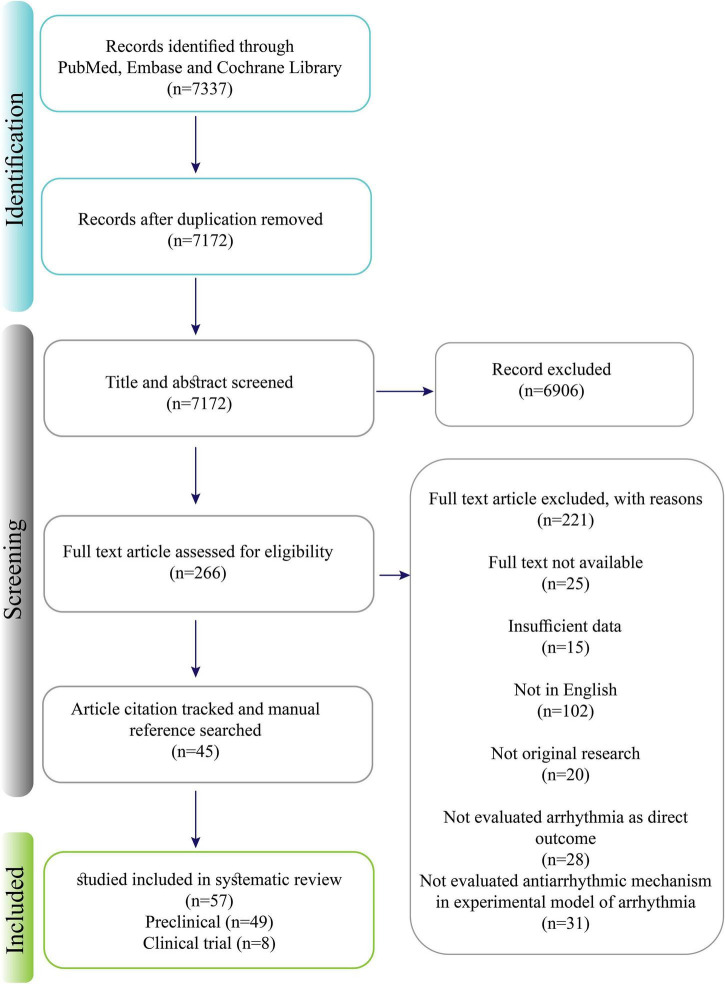
Prisma chart of the manuscript selection process.

Figure 1 shows the process of study selection. Among a total of 7337 primarily retrieved articles, 49 preclinical and eight clinical studies were finally included. Out of the 49 preclinical studies, four (8.1%) corresponded to plant extract, 10 (20.4%) to multi-component herbal preparations, and 35 (71.4%) to phytochemicals. One clinical study focused on phytochemicals, and the other seven were devoted to multi-component herbal preparations. The antiarrhythmic effects were mostly mediated by affecting K^+^ channels (class III), followed by modulating Ca^2+^ channels (IV), upstream target pathways (class VII), Na_*v*_ channels (class I), gap junction channels (class VI), and autonomic receptors (class III). All the studies that investigated the inhibitory effects of herbal medicine on HCN (class 0) and mechanosensitive TRPC (class V) channels were designed in physiological conditions, and we found no study that evaluated such effects in the arrhythmia models. The detailed characteristics of the final selected studies are summarized in Tables 1–4.

**TABLE 1:** Plant extracts with antiarrhythmic effects and their mechanism of action.

Plant extracts							
Scientific name of plant(s)	Type of extract and active constituents	Study design/Model	Duration	Dosage/Intervention	Results	Mechanism (class)	References
*Arnebia euchroma (Royle) I. M. Johnst.*	Root decoction	male SD rats/ACh/CaCl_2_ induced arrhythmia	7 days	0.09 g/100 g/day PO	↓ AF susceptibility and occurrence	VII	(80)
*Nardostachys chinensis Batalin*	Rhizome paste	Male SD rats/I/R-induced arrhythmia	30 days	600 mg/kg/day PO	↓ incidence of VF, VT, and PVCs	VI	(66)
*Rhodiola crenulata (Hook.f. and Thomson) H. Ohba*	Water extract of *R. crenulata* and its main compound Salidroside	New Zealand White rabbits/I/R-induced arrhythmia	4 weeks	125, 250, 500 mg/kg extract and 9.5 mg/kg salidroside every 2 days PO	Inhibition of VA inducibility in HF rabbits	III, IV, VII	(41)
	Water extract of *R. crenulata*	Rabbit model of HF	2 weeks	270 mg/kg/day orally	↓ AF inducibility		(42)

Ach, acetylcholine; AF, atrial fibrillation; HF, heart failure; I/R, ischemia/reperfusion; PO, per os (by mouth); PVC, premature ventricular contractions; SD, Sprague Dawley; VA, ventricular arrhythmia; VF, ventricular fibrillation; VT, ventricular tachycardia; ↓, decrease.

**TABLE 2:** Multi-component herbal preparations with antiarrhythmic effects and their mechanism of action.

Multi-component herbal preparations						
Name	Study design	Duration	Dosage/Intervention	Outcomes	Mechanism (class)	References
Danqi soft capsule (*Salvia miltiorrhiza Bunge* and *Panax notoginseng (Burkill) F. H. Chen*)	Male SD rats/I/R-induced arrhythmia	4 weeks	0.6, 0.9, and 1.2 g/kg/day gavage	↓ VT induction ↓ Arrhythmia severity scores	VI, VII Other potential mechanism: V (63)	(62)
Dingji fumai decoction (*dried rhizome of Ligusticum striatum DC, seed and dried fruit of Ziziphus jujube Mill, dried twig of Cinnamomum cassia (L.) J. Presl, Albizia julibrissin Durazz bark, the dried root of Polygala tenuifolia Willd, Glycyrrhiza glabra L, Poria cocos, Os draconis, Ostrea Gigas thunberg*)	Male SD rats/I/R-induced arrhythmia	14 days	17.6 g/kg/day gavage	↑ onset time of VA ↓ duration of VA	I	(37)
Fumai granule (the dried root of Panax ginseng C. A. Meyer, Bitter Ginseng, *Ophiopogon japonicas (Thunb.) Ker Gawl.*, *Schisandra chinensis (Turcz.) Baill.*, *Angelica sinensis (Oliv.) Diels*, *Glycyrrhiza uralensis Fisch*)	Male SD rats/Programmed electrical stimulation-induced AF	4 weeks	500 mg/kg/day gavage	↓ inducibility of AF ↓ mean AF episode duration	VI, VII	(75)
Ping-Lv-Mixture (*Glycyrrhiza inflate Batalin*, *Sophora flavescens Aiton*, *Belamcanda chinensis (L.) DC.*, *Sophora tonkinensis Gagnep, Schisandra chinensis (Turcz.) Baill.*)	Male SD rats/I/R-induced arrhythmia	7 days	0.04, 0.2, and 1 g/kg/day gavage	↓ the incidence and duration of PVB, VT and VF	VII	(91)
Shensong Yangxin capsule (*Panax ginseng C. A. Mey*, *Ophiopogon japonicas (Thunb.) Ker Gawl*, *Nardostachys grandiflora DC*, *Taxillus chinensis (DC.) Danse*, *Ziziphus jujube Mill*, *Cornus officinalis Siebold and Zucc*, *Salvia miltiorrhiza Bunge*, *Paeonia lactiflora Pall*, *Schisandra sphenanthera Rehder and E. H. Wilson*, *Polypodiodes chinensis (H. Christ) S. G. Lu*, *Eupolyphaga sinensis*, *Coptis chinensis* Franch.)	Male SD rats/Programmed electrical stimulation-induced AF	4 weeks	600 mg/kg/day gavage	↓ rate of AF inducibility ↓ mean AF episode duration	III, VII Other Potential mechanisms: 0, V (16, 63)	(15)
	Male SD rats/I/R-induced arrhythmia	7 days	1.8 g/kg/day intragastrical	↓ incidence of VT and VF ↓ arrhythmia severity scores		(57)
Tongguan capsule (*Astragalus mongholicus Bunge*, *Salvia miltiorrhiza Bunge*, *Ophiopogon japonicus* (Thunb.) Ker Gawl, *Hirudo medicinalis*)	Male SD rats/I/R-induced arrhythmia	4 weeks	0.62 or 1.24 g/kg/day gavage	↓ VT induction ↓ arrhythmia scores	VI Other potential mechanisms: V (63)	(74)
WenXin KeLi (*Nardostachys chinensis Batalin, Codonopsis, Notoginseng*, Rhizoma Polygonati extracts, and amber)	canine right atrial/ACh-induced AF	30–60 min	5 g/L Perfusion	preventing the induction of AF, and termination of persistent AF	I, III, IV	(34)
	Rat/I/R-induced arrhythmia	3 weeks	8 g/kg/day gavage	↓incidence of VT and VF ↓ number VEBs/min episodes ↓ VA severity		(56)
Yindanxinnaotong capsule (the leaf of *Ginkgo biloba* L, the root and rhizome of *Salvia miltiorrhiza Bunge*, aerial part of *Erigeron breviscapus* (Vaniot) Hand.-Mazz, fruit of *Crataegus pinnatifida* Bunge, the root and rhizome of *Panax notoginseng* (Burkill) F. H. Chen, aerial part of *Gynostemma pentaphyllum* (Thunb.) Makino, the bulb of *Allium sativum* L, l-borneolum)	Male SD rat isolated heart/I/R-induced arrhythmia	110 min	(Ginkgo biloba extract: 0.025 mg/mL and ethanol extract of salvia miltiorrhiza: 0.013 mg/mL and mixed combination of other com-ponent: 0.05 mg/mL) Perfusion	↓incidence of VT and VF ↓duration of arrhythmias	VII Other potential mechanisms: V (63)	(92)

min, minutes; PVB, premature ventricular beat; VEB, ventricular ectopic beats; other abbreviation as in Table 1.

**TABLE 3:** Phytochemicals with antiarrhythmic effects and their mechanism of action.

Phytochemicals						
Phytochemical	Study design	Duration	Dosing/Intervention	Outcomes	Mechanism (class)	References
**Alkaloids**						
Berberine	Male SD rats/Aconitin-induced arrhythmia	7 days	8 and 16 mg/kg/day intragastric	↓ occurrence of VT, VF and premature ventricular bigeminy, trigeminy ↓mortality	III Other potential mechanism: 0 (17)	(44)
	Male SD rats/I/R-induced arrhythmia	7 days	100 mg/kg/day PO	↓ duration of arrhythmias ↓ arrhythmias score		(45)
Liriodenine	Male Wistar rats isolated heart/I/R-induced arrhythmia	Acute administration	0.3, 1, and 3 μM Perfusion	Conversion of the tachyarrhythmias to normal sinus rhythm	I, III	(21)
Oxymatrine	Male SD rats/I/R-induced arrhythmia	Acute administration	5, 10, and 20 mg/kg IV injection	↑ onset time of VA ↓ duration of VA ↓ arrhythmia severity score	I, III, IV	(50)
	Male Wistar rat/I/R-induced arrhythmia	Acute administration	3, 10, and 30 mg/kg IV injection	↓ duration of VA ↑ onset time of VA ↓ susceptibility to VA		(19)
Matrine	Male Wistar rat/I/R-induced arrhythmia	7 days	15 and 30 mg/kg/day Intragastric	↓ duration of arrhythmias in 30 mg group ↓ arrhythmia severity score ↓ incidence of sudden death	III	(51)
**Flavonoids**						
Acacetin	Mongrel dog/Vagotonic-induced AF	Acute administration	2.5, 5, and 10 mg/kg Intradeudenal injection	↓ incidence of AF ↓ sustained AF	III	(43)
Grape seed proanthocyanidin extracts	New Zealand rabbits/I/R-induced arrhythmia	21 days	100 and 200 mg/kg/day of proanthocyanidin PO	↓ incidence of VF and VT	VI	(68)
	Male Wistar rats/I/R-induced arrhythmia	4 weeks	200 mg/kg/day of proanthocyanidin PO	↓ incidence of VF and VT		(67)
VCF flavonoid extract of *Viscum coloratum*	Wistar rats/Aconotine-induced arrhythmia	Acute administration	15 and 75 mg/kg IV injection	↓ susceptibility to PVC, VT, and VF	IV	(60)
Bilberry Anthocyanins	Male Wistar rats isolated heart/I/R-induced arrhythmia	10 min	0.01, 0.1, 1, 5, 10, 25, and 50 mg/L Perfusion	↓ duration of arrhythmias in 0.1, 1, and 5 mg/L group ↓ incidence of VT and VF in 0.1, 1, and 5 mg/L group	VII	(88)
Curcumin	Rabbit isolated heart/I/R-induced arrhythmia	30 min	30 μmol/L Perfusion	↓ the incidence and average duration of the VT and VF	I, III, IV	(23)
Hesperidin	Male SD rats/I/R-induced arrhythmia	15 days	100 mg/kg PO	↓ incidence and duration of VT and VF ↓ severity of the arrhythmia	VII	(82)
	Male Wistar rats isolated heart/I/R-induced arrhythmia	150 min	1, 2.5, and 5 μg/ml	↓ incidence VEB and VT ↓ duration of VT		(83)
Total flavonoids from *Hypericum attenuatum* Fisch. ex Choisy	Male SD rats/I/R-induced arrhythmia	7 days	100 mg/kg/day Gavage	↑ onset time of arrhythmia ↓ duration of arrhythmia	III, IV	(40)
Troxerutin	Wistar rats isolated heart/I/R-induced arrhythmia	30 days	150 mg/kg/day Gavage	↓ number of PVC ↓ incidence of VF ↓ duration of VT and VF ↓ severity of arrhythmias	VII	(84)
**Lignin**						
Magnolol	Male SD rats/I/R-induced arrhythmia	Acute administration	0.1, 0.01, and 0.001 μg/kg IV injection	↓ incidence of VT and VF ↓ Duration of VT and VF	VII	(85)
			0.2 and 0.5 μg/kg IV injection	↓ incidence of VF ↓ Duration of VT and VF		(86)
Arctigenin	Male SD rats/I/R-induced arrhythmia	7 days	12.5, 50, and 200 mg/kg/day Intragastric	↓ numbers of episodes of VT and VF ↓Incidence and duration of VT and VF ↓severity of arrhythmias	I, IV, VII	(25)
	Wistar rats Aconitine-induced arrhythmia	8 min	2 and 1 mM IV infusion	delayed the onset time of arrhythmias		(24)
Cinnamophilin	Rats isolated heart/I/R-induced arrhythmia	Acute administration	1 to 10 μM Perfusion	Conversion of tachyarrhythmia to sinus rhythm	I, III, IV	(26)
**anthraquinones**						
Barbaloin	Rabbit isolated heart/I/R-induced arrhythmia	90 min	200 μmol/L Perfusion	delayed the onset time ↓incidence of VT and VF	I, IV	(27)
**Stilbenoids**						
Resveratrol	Male SD rats/I/R-induced arrhythmia	Acute administration	2.3 × 10^–7^, 2.3 × 10^–6^, and 2.3 × 10^–5^ g/kg IV injection	↓ incidence and duration of VT and VF	I, III, IV	(28)
	Male SD rats/I/R-induced arrhythmia or electrical burst-induced arrhythmia	28 days	5 mg/kg/day PO	↓ incidence of VT and VF		(29)
	Wistar rats/Aconitine-induced arrhythmia	Acute administration	5, 15, and 45 mg/kg IV injection	↓ susceptibility to PVC and VT and VF ↓ duration of VA		(30)
	Guinea pigs/Ouabain induced arrhythmia	Acute administration	5, 15, and 45 mg/kg IV injection			
	Male SD rats isolated heart/I/R-induced arrhythmia	2 h	3–100 μM Perfusion	Reversed the VT-induced by I/R		(33)
**Terpenoids**						
Andrographolide	New Zealand white rabbits/Aconitine-induced arrhythmias	5 min before aconitine injection	10 mg/kg IV injection	↑ aconitine cumulative dosage required to induce PVC, VT, and VF ↓ mortality ↓ incidence of VA	I, IV	(18)
Ginsenoside Rb1	Mice/CaCl_2_/ACh-induced AF	Acute administration	30 or 50 mg/kg IP injection	Significant inhibition of AF	IV	(59)
	Mice/Chloroform-induced-VF			Significant inhibition of VF		
	SD rats/BaCI_2_-induced VT			Significant inhibition of VT		
Linalool	Male SD rats/I/R-induced arrhythmia	7 days	50 or 100 mg/kg/day NI	↓ the arrhythmia scores ↓ frequency and duration of VA	VI	(72)
Oleanic acid	Wistar male rats/CaCl_2_ or adrenalin or I/R-induced arrhythmias	Acute administration	40 mg/kg IP injection	↓ incidence of VT, VF and PVB	II	(39)
Ursolic acid						
Panax notoginseng saponin	Male SD rats/ACh/CaCl_2_-induced AF	7 days	100 and 150 mg/kg IP injection	↓ duration of AF	IV	(87)
Sasanquasaponin	Male ICR mice/I/R-induced arrhythmias	Acute administration	0.1, 0.2, and 0.4 mg/kg IV injection	↓ incidence VT, VF, VBP and salvos Acute treatment effect on SA arrhythmias and VT	III	(52)
	Male ICR mice isolated heart/I/R-induced arrhythmias	Acute administration	0.1 μM Perfusion	↓ incidence VT, VF, VBP and salvos		
Tanshinone IIA	Male Wistar rats/I/R-induced arrhythmia	90 days	10 mg/kg/day NI	↓ the duration of arrhythmias ↓ the incidence of VT/VF ↓ arrhythmia severity score	III	(53)
**Sulfur compounds**						
Allicin	Male Wistar rats/BaCI_2_-induced arrhythmia	28 days	4, 8, and 16 mg/kg/day IP injection	↑onset time of arrhythmias ↓ score of arrhythmias	III, IV	(54)

IP, Intraperitoneal; IV, intravenous; h, hours; NI, no information; VBP, ventricular premature beats; other abbreviations as in Tables 1, 2.

**TABLE 4:** Summary of the clinical studies investigating the efficacy and safety of phytochemicals or multi-component herbal preparations on cardiac arrhythmias.

Clinical trials					
Natural source	Study design	Intervention/Dosing	Duration	Outcomes	References
Dingji Fumai Decoction	Randomized, double-blind, controlled trial in 136 symptomatic patients with PVC	1200 mg DFD TDS combined with metoprolol 12.5 mg bid or placebo plus metoprolol 12.5 mg bid	12 weeks	↓ number of PVC ↓ severity of heart palpitation and the functional condition	(93)
	Randomized, positive controlled parallel-group trial in 92 patient	200 ml DFD TDS combined with metoprolol 12.5 mg bid or metoprolol 12.5 mg bid alone in the control group	4 week	↓TCM syndrome score ↓ number of PVC	(36)
Shengmai injection (*Panax ginseng* C. A. Mey. and *Ophiopogon japonicas* (Thunb.) Ker Gawl.)	A controlled trial in 351 AF patient	100 ml IV infusion of shenmai injection once a day combined with amiodarone 150 mg in 10 min then followed by 1 mg/min and 0.5 mg/min or placebo plus amiodarone	48 h	↑rate of success in the treatment of AF ↓ time to treatment of AF	(94)
Baicalin (flavonoid isolated from the dried root of *Scutellaria baicalensis Georgi*)	A controlled trial in 60 patients with acute aconitine poisoning	450 mg of baicalin *via* IV infusion followed by Ringer or dextrose 5% plus KCl and Vit C daily with a rate of 3 ml/min and the same treatment without baicalin for control group	48 h	↓ recovery time from PVC, auricular flutter, AF, sinus bradycardia and auriculoventricular block Significantly more efficacious than the conventional treatment.	(95)
Shensongyangxin	Randomized, double-blind, placebo, and positive controlled trial in 411 in CHF Patients with Frequent PVCs	4 × 1.6 g SSYX capsule TDS orally in combination with mexiletine 450 mg or placebo plus the same dose of mexiletine	8 week	↓ number of PVCs in organic and non-organic heart diseases ↑ rate of effectiveness in treatment compared to mexiletine	(96)
	Randomized, double-blind, placebo, and positive controlled trial in 769 patients with organic or non-organic heart disease with frequent PVCs	4 × 1.6 g SSYX capsule tid orally in combination with mexiletine 450 mg or placebo plus the same dose of mexiletine	12 week	↓ number of PVCs compared to placebo ↑ rate of effectiveness in treatment compared to mexiletine ↑ improvement in cardiac function	(97)
Wenxin Keli	Randomized, double-blind, a placebo-controlled trial in 1071 patients with frequent PVCs	9 g of Wenxin Keli orally TDS or placebo	4 weeks	↓ number of PVCs in Wenxin Keli group compared to placebo	(98)
Xin Su Ning	Randomized, double-blind, placebo, and positive controlled trial in 829 patients with PVCs	XSN four capsules, 0.48 g per capsule, 50 mg mexiletine, or placebo groups	4 weeks	↓ number of PVCs ↑ improvement rate of PVC-related symptoms	(99)

bid, bis in die (twice a day); CHF, congestive heart failure; DFD, Dingji Fumai Decoction; TCM, traditional Chinese medicine; TDS, ter die sumendus (3 times a day); SSYX, Shensongyangxin; Vit, vitamin; XSN, Xin Su Ning; other abbreviations as in Tables 1–3.

**FIGURE 2:**
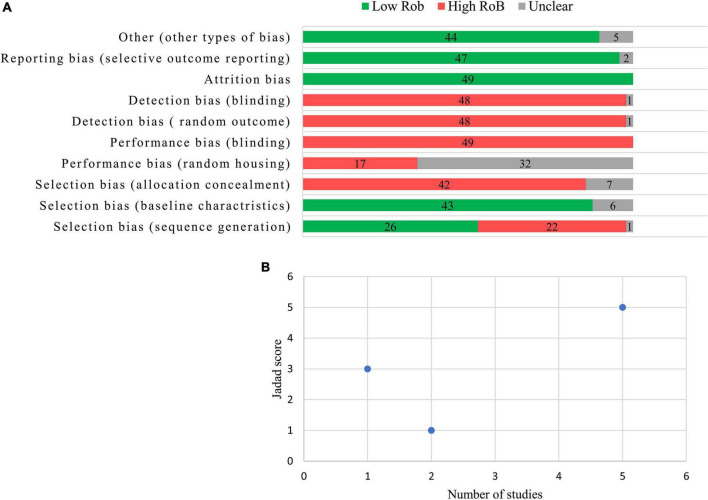
Results from the risk of bias assessment according to the Systematic Review Centre for Laboratory Animal Experimentation (SYRCLE) authors’ judgment about each risk of bias item **(A)** and the quality assessment of clinical trials according to the Jadad score **(B)**.

Figure 2A presents the quality assessment of the preclinical studies based on SYRCLE’s RoB tool. Twenty-eight studies (53.0%) mentioned “random” in sequence generation, but none of the studies explained the randomization method. The bias risk assessment results of “baseline characteristics” in six studies were unclear because there was no information about animal samples in the experimental and control groups. No study reported the “allocation concealment” clearly. In the experiment implementation stage, almost all of the studies did not mention “random housing to animals,” “blinding in the study design or assessments of outcomes,” and “random selection of animals for outcome assessment.” All studies reported low bias in “addressing the incomplete outcome data,” “selective outcome reporting,” and other biases. Figure 2B presents the quality assessments of the clinical studies based on the Jadad score. Five studies involved a double-blind process and were judged to be of high quality (Jadad score = 5). Two studies were not randomized clinical trials and were judged to be of poor quality (Jaded score = 1). One study with a Jaded score of 3 had moderate quality.

### Antiarrhythmic mechanisms of medicinal plants: preclinical studies

**FIGURE 3:**
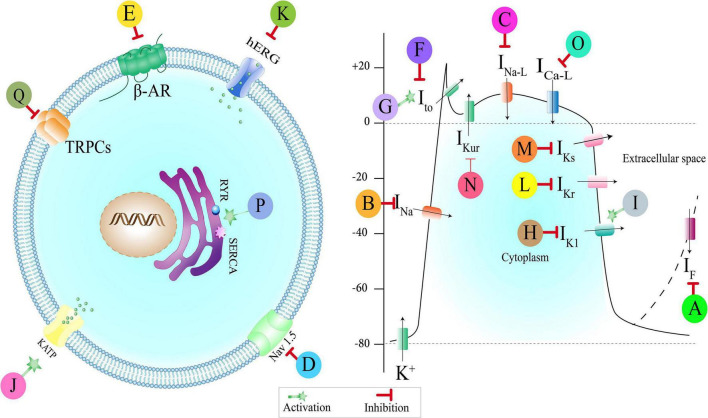
Medicinal plants, multi-component herbal preparations, and phytochemicals with an antiarrhythmic effect based on their effect on ionic channels. Some of them have more than one mechanism. Class (0): A: Shensong Yangxin capsule, Ginkgo biloba. Class (I): B: Andrographolide, Oxymatrine, Liriodenine, Arctigenin, Resveratrol, C: Wenxin Keli, Curcumin, Barbaloin, D: Dingji Fumai decoction, Xin Su Ning. Class (II): E: olea europaea. Class (III): F: Wenxin Keli, Shensong Yangxin capsule, Allicin, Resveratrol, Cinnamophilin, Acacetin, G: Oxymatrine, H: Berberin, Shensong Yangxin capsule, Oxymatrine, I: Tanshinone IIa, *Rhodiola* spp., J: Sasanquasaponin, *Total flavonoids from Hypericum attenuatum*, K: Xin Su Ning, L: curcumin, M: Resveratrol, N: Acacetin. Class (IV): O: Wenxin Keli, Allicin, Total flavonoids from *Hypericum attenuatum*, flavonoid extract of *Viscum coloratum*, Ginsenoside Rb1, Oxymatrine, Curcumin, Arctigenin, Cinnamophilin, Barbaloin, Resveratrol, *Rhodiola* spp., P: *Rhodiola* spp. Class (V): Q: Salvianolic acid-B. I_F_: Pacemaker current, I_Na_: Sodium currents, I_Na–L_: Late sodium currents, Nav: voltage-gated sodium channel, I_to_: Transient outward current, I_K1_: Inward rectifier current, K_ATP_: ATP activated K^+^ channels, hERG: Human Ether-a-go-go-related Gene (hERG) Potassium Channel, I_Kr_: Rapid delayed rectifier current, I_Ks_: Slow delayed rectifier current, I_Kur_: Ultrarapid delayed rectifier currents, I_Ca–L_: L-type-calcium current, RYR: Ryanodine receptors, SERCA: Sarcoendoplasmic reticulum calcium transport ATPase, TRPCs: Transient receptor potential canonical channels, β-AR: Beta-adrenergic receptor.

**FIGURE 4:**
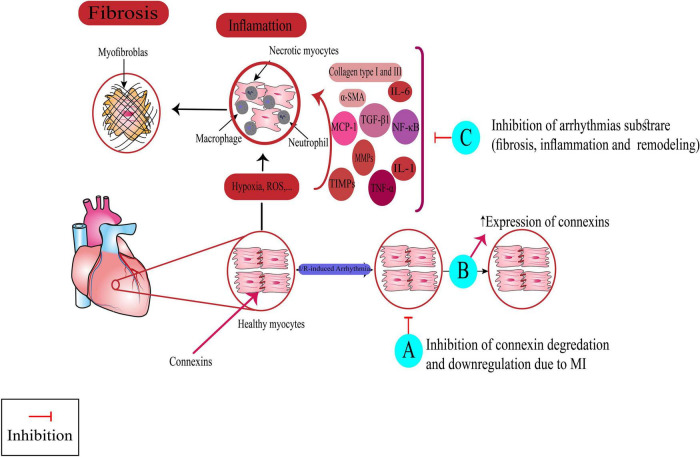
Targeting non-ionic mechanism for suppressing arrhythmias by medicinal plants, multi-component herbal preparations, and phytochemicals. Some of them have more than one mechanism. Class (VI): A: *Nardostachys chinensis*, Linalool, B: Danqi soft capsule, Fumai granule, Tongguan capsule, Grape seed proanthocyanidin. Class (VII): C: *Arnebiae euchroma, Rhodiola spp.*, Danqi soft capsule, Ping-Lv-Mixture, Shensong Yangxin capsule, Fumai granule, Bilberry Anthocyanins, Hesperidin, Troxerutin, Magnolol, *Panax notoginseng* saponins, Arctigenin, Yindanxinnaotong capsule. Nrf2: Nuclear factor erythroid 2-related factor 2, TGF-β1: transforming growth factor-beta1, MCP-1: monocyte chemoattractant protein-1, TNF-α: tumor necrosis factor-α: TNF-α, TIMPs: Tissue inhibitors of metalloproteinases, MMPs: Matrix metalloproteinases, IL: interleukin, α-SMA: Smooth muscle alpha-actin, NF-κB: nuclear factor- κB, I/R: Ischemia-reperfusion, ROS: reactive oxygen species.

Medicinal plants found in our review exerted their antiarrhythmic effects *via* different mechanisms. Here, we discussed the experimental studies in which antiarrhythmic effects of plant extract, phytochemicals, and multi-component herbal preparations have been investigated alongside their underlying antiarrhythmic mechanisms. Several other plants with antiarrhythmic effects whose probable mechanisms of action have been explored in another independent study on physiologic models were not discussed in the body of the article and merely summarized in the Supplementary Tables 1–3. Figures 3, 4 illustrate a schema of the mechanisms affected by such compounds to manage cardiac arrhythmias.

#### Class (0): HCN channel-mediated pacemaker current blocker

The hyperpolarization-activated cyclic nucleotide-gated (HCN) channel family is composed of transmembrane channels with a single pore, among which HCN2 and HCN4 are the predominant isoforms of the heart. The HCN channel-mediated pacemaker current (*I*_*f*_) is essential for the generation and conduction of cardiac impulses. They are highly expressed in the sinoatrial node (SAN), and their inhibition decreases the spontaneous activity (automaticity) of the sinoatrial node (SAN) by affecting phase 4 of depolarization, thereby controlling the increased heart rate (7, 10). Although the expression level of HCN channels is very low in normal myocardium, it seems to be increased in pathologic conditions such as ischemic cardiomyopathy and congestive heart failure. In such pathologic settings, *I*_*f*_ may be responsible for inappropriate automaticity in the ventricular cardiomyocytes, leading to ventricular premature complexes that may generate ventricular arrhythmias. Therefore, it appears that inhibiting this current with medications in this class, like ivabradine, can diminish arrhythmia in such pathologic conditions, as preclinical studies have indicated. In the context of ischemia, the antiarrhythmic effects may be the result of reduced heart rate, resulting in energy preservation and mitigation of ischemia-induced electrophysiological consequences. In the setting of congestive heart failure, the antiarrhythmic effects may be attributable to the inhibition of HCN channels, which consequently reduces the proarrhythmic pathological automaticity in ventricular cardiomyocytes (11, 12).

Regarding this class of AADs, our review found no study that, besides the antiarrhythmic effects of the plant, also examined concurrently such mechanism of action for that. However, we found a number of plant extracts and multi-component herbal preparations, as described below and summarized in Supplementary Table 1, whose inhibitory effects on *I*_*f*_ have been identified in an *in vitro* model of pacemaker cells in some studies, while their antiarrhythmic effects have been evaluated in some other separate studies.

*Ginkgo biloba* L. extract and one of its main active ingredients, namely bilobalide, were shown to have antiarrhythmic effects in rat models of ischemia-reperfusion (I/R) injury (13, 14). One study by Satoh et al. (15) indicated that this plant diminishes the pacemaker activity in SAN, thereby exerting a negative chronotropic effect in a concentration-dependent manner. This study also found that modulating multiple currents in SAN, including but not limited to *I*_*f*_, is probably responsible for such an effect. Thus, blockade of *I*_*f*_ might be one of the antiarrhythmic mechanisms for this plant, probably exerting through reducing heart rate, which leads to energy preservation and a reduction in ischemia-induced electrophysiological consequences. However, further studies in experimental models of cardiac arrhythmias are warranted to know the exact effect of this plant on *I*_*f*_ in pathologic conditions.

Shensong Yangxin (SSYX) capsule is a traditional Chinese medicine (TCM) consisting of 12 medicinal plants, approved clinically for its antiarrhythmic effects in China through affecting multiple channels in cardiac pacemaker cells (16). One study by Sun et al. (17) found that SSYX can inhibit the *I*_*f*_ *via* reducing the expression of HCN channel genes. Also, our literature search found that one of the active ingredients in the SSYX capsule, namely berberine, can act as an *I*_*f*_ blocker, which might contribute to its antiarrhythmic efficacy (18). Thus, we can expect that the antiarrhythmic effects of SSYX might be, at least in part, due to the inhibition of *I*_*f*_, similar to that seen in class (0) AADs. However, there is no study that has examined this mechanism in an arrhythmia model, and this issue should be considered in future studies.

#### Class (I): Voltage-gated Na^+^ channel blockers

Voltage-gated Na^+^ (Na_*v*_) channels are transmembrane proteins composed of a pore-forming subunit and auxiliary subunits that are extensively expressed throughout the atrial, Purkinje conducting, and ventricular cardiomyocytes, where they are responsible for the rapid upstroke of the cardiac action potential (AP) and the rapid conduction of impulses through cardiac tissue. Consequently, dysfunction in Na_*v*_ channel current (*I*_*Na*_) plays an important role in the formation of cardiac arrhythmias (19, 20). Na_*v*_ channel blockers comprise the class (I) AADs, which itself is divided into four subclasses (Ia, Ib, Ic, and Id), all of which generally influence the upstroke and duration of AP (7). Below, we described several phytochemicals and multi-component herbal preparations whose antiarrhythmic effects are exerted through inhibiting Na_*v*_ channels like the action of class (I) AADs.

Andrographolide is a labdane diterpenoid isolated from the leaves and roots of *Andrographis paniculata* (Burm.f.) Nees with several cardioprotective effects. Zeng et al. (21) conducted an *in vivo* experiment with an aconitine-induced arrhythmia animal model as well as *in vitro* assays, using rabbit left ventricular and left atrial myocytes to confirm their hypothesis that andrographolide has antiarrhythmic effects through modulation of cardiac ion channels. They showed that 20 μM andrographolide suppressed the aconitine-induced arrhythmias in the *in vivo* rabbit model. In the *in vitro* analyses, Andrographolide shifted the *I*_*Na*_ and *I*_*Ca*_ inactivation curves toward negative membrane potential and expedited the inactivation process, thereby inhibiting the entry of Na^+^ and Ca^2+^ into the cardiomyocyte. In addition, an electrophysiological evaluation showed a significant decrease in AP duration and maximal upstroke velocity (V_*max*_) of phase 0 depolarizarion as well as the cellular mechanisms underlying cardiac arrhythmias, including delayed afterdepolarizations (DADs)-induced triggered activities induced by isoproterenol and high Ca^2+^ administration. Thus, the authors indicated that andrographolide suppressed aconitine-induced arrhythmias *via* the inhibition of *I*_*Na*_ and *I*_*Ca*_, similar to that seen in class (I) and class (IV) of AADs.

Oxymatrine is an alkaloid compound isolated from *Sophora flavescens* Aiton roots and demonstrated to have cardioprotective effects. Runtao et al. (22) investigated the effect of oxymatrine on aconitine-induced and coronary artery ligation-induced arrhythmias in rats and its fundamental mechanisms. They indicated that oxymatrine (3, 10, and 30 mg.kg^–1^) could prevent these arrhythmias in a concentration-dependent manner. In *in vitro* analysis of isolated rat cardiomyocytes, oxymatrine significantly inhibited *I*_*Na*_ and *I*_*Ca*_, similar to the action of class (I) and class (IV) of AADs, which could explain the observed antiarrhythmic effects in this study.

Liriodenine is an aporphine-type alkaloid extracted from *Fissistigma glaucescens* (Hance) Merr and shown to have beneficial effects on the cardiovascular system (23). The antiarrhythmic potential of liriodenine was investigated by Chang et al. (24), who assessed the effect of this active compound in an *ex vivo* model of I/R-induced arrhythmias in the Langendorff perfused rat heart. They found that liriodenine with a half maximal effective concentration (EC50) of 0.3 μM suppressed the I/R-induced arrhythmias in a dose-dependent manner. Additionally, in a concentration-dependent manner, liriodenine increased AP duration and reduced the V_*max*_ of phase 0 depolarization in isolated ventricular myocytes from rats. They also showed in the whole-cell patch-clamp recording that such electrophysiological effects were due to the inhibition of *I*_*Na*_ and transient outward K^+^ current (*I*_*to*_), respectively. Such effects can be likened to the actions of class (I) and class (III) of AADs, respectively.

Curcumin, a flavonoid found in *Curcuma longa* L rhizomes, has shown to have a wide range of cardioprotective effects (25). Song et al. (26) evaluated the antiarrhythmic effects of curcumin (30 mM) and indicated its ability to reduce the incidence and duration of I/R-induced ventricular tachyarrhythmias (VT) and ventricular fibrillation (VF) in Langendorff-perfused rabbit hearts. In *in vitro* analysis, curcumin reduced the AP duration and suppressed early afterdepolarization (EAD) and Ca^2+^-induced DADs through inhibiting multiple ion channel currents, specifically late Na^+^ channel current (*I_Na–L_*) and, to some extent, voltage-gated L-type Ca^2+^current (*I*_*Ca–L*_) and transient Na^+^ current (*I*_*Na–T*_). Moreover, in this study, curcumin inhibited rapid delayed rectifier K^+^ current (*I*_*Kr*_), which although such inhibition can cause the AP duration to lengthen, because of concurrent inhibition of *I*_*Na–L*_ and *I*_*Ca–L*_, curcumin shortened the AP duration altogether. Thus, curcumin exerts its antiarrhythmic effects like the action of class (I), class (III), and class (IV) of AADs.

Arctigenin (ATG) is a lignan isolated from the dried fruit of *Arctium lappa* L., shown to be effective in combating cardiac arrhythmias (27, 28). Zhao et al. (27) showed that 100 μM ATG markedly delayed the onset of aconitine-induced arrhythmia in the rat model. In the *in vitro* analysis, ATG shortened the AP duration, increased the *I*_*to*_, and produced inhibitory effects on *I*_*Na*_ and *I*_*Ca–L*_. Thus, the authors suggested that the antiarrhythmic effect of ATG may be mediated by modulating multiple ion channels.

Cinnamophilin, a lignan extracted from *Cinnamomum philippinense* (Merr.) C. E. Chang was found to have protective effects against cardiac arrhythmias. Su et al. (29) demonstrated that administration of cinnamophilin (1 to 10 μM) reversed VTs induced by I/R into the sinus rhythm in isolated heart rat preparations. They also found that such an effect may be partly related to the inhibition of *I*_*Na*_, leading to a reduction in V_*max*_, similar to the action of class (I) AADs. Cinnamophilin was also found to have inhibitory effects on *I*_*to*_ and *I*_*Ca*_, which may exert antiarrhythmic effects like the action of class (III) and class (IV) of AADs. *I*_*to*_ inhibition leads to an increase in AP duration, which may generate proarrhythmic effects similar to those found with class (III) AADs. However, concurrent inhibition of the *I*_*Ca*_ by cinnamophilin may reduce this prolongation, resulting in fewer proarrhythmic effects.

Barbaloin is an anthraquinone-type phenolic compound with multiple biological activities, extracted from *Aloe* spp. Using isolated rabbit ventricular myocytes as an *in vitro* animal model, Cao et al. (30) discovered that after the treatment with barbaloin (200 μM), the onset time of aconitine-induced VT and VF was prolonged, and their incidence was decreased. The cellular electrophysiological evaluation showed that barbaloin (100 and 200 μM) could markedly shorten the AP duration and terminate *Anemonia sulcata* toxin II-induced EADs and Ca^2+^-induced DADs in isolated myocytes in a dose-dependent manner. These electrophysiological changes, together with inhibition of *I*_*Na–L*_ and *I*_*Ca–L*_ discovered in this study, may explain the antiarrhythmic effects of barbaloin, which are similar to the action of class (I) and class (IV) of AADs.

Resveratrol is a phytoalexin isolated from some plants such as *Vitis* and *Vaccinium* species. Several animal studies have proved the protective effects of resveratrol against cardiac arrhythmias (31–36). Among them, Chen et al. (36) evaluated the antiarrhythmic effects of resveratrol and its ability to change *I*_*Na*_ in a rat model of isolated myocytes. In this study, resveratrol eliminated the I/R-induced ventricular arrhythmias, reduced V_*max*_ and prolonged the AP duration and effective refractory period (ERP), all of which were shown to be related to the inhibition of *I*_*Na*_, *I*_*to*_ and sustained outward K^+^ currents (*I*_*ss*_) in cellular electrophysiological recordings. Such effects are similar to the actions of class (I) and class III of AADs. Resveratrol was also shown to have a partial inhibitory effect on *I*_*Ca–L*_, which can explain the prolonged atrioventricular (AV) nodal conduction observed in this study.

Wenxin Keli (WXKL) is a TCM composed of a mixture of five natural compounds (37). Burashnikov et al. (37) investigated the antiarrhythmic effects of WXKL in an *ex vivo* model of isolated canine arterially perfused right atrial preparations. They found that WXKL can effectively suppress acetylcholine (ACh)-induced atrial fibrillation (AF) and prevent its induction *via* affecting the electrophysiological properties of Na_*v*_ channels. In this study, WXKL increased the ERP, induced rate-dependent post-repolarization refractoriness, and decreased V_*max*_, predominantly in the atria. These atrial-selective properties are similar to those seen in Ranolazine, an antiarrhythmic drug belonging to class Id, shown to be effective in the management of AF and *I*_*Na–L*_-related tachyarrhythmias (7, 38).

Dingji Fumai decoction (DFD), a herbal mixture composed of nine different herbs, has been traditionally prescribed for treating palpitation (39). Liang et al. (40) investigated the effect of DFD on BaCl_2_-induced and aconitine-induced arrhythmias in rats. They found that pretreatment with DFD (17.6 g.kg^–1^. once a day) for 2 weeks could reduce the duration of ventricular arrhythmias and delay their initiation mainly through four mechanisms: (1) dose-dependent inhibition of Na_*v*_1.5 current, (2) modulating Na^+^-K^+^-ATPase activity, (3) modulating gap junctions, and (4) antioxidant properties; The former is similar to that effect exerted by class (I) AADs (7).

#### Class (II): Autonomic inhibitors and activators

Autonomic imbalance is known as a key player in the development of CVDs. The parasympathetic and sympathetic branches of the autonomic nervous system exert various effects on the human myocardium through the activation of different G protein-coupled receptors that mediate the interplay between autonomic nervous system function and the cardiovascular system and can be targeted by multiple drugs with the purpose of preventing and treating CVDs (41). According to the modernized classification of AADs, autonomic activators and inhibitors belong to class II, and are categorized into five subclasses (IIa, IIb, IIc, IId, and IIe) (7).

*Olea europaea* L., commonly known as olive, has demonstrated antiarrhythmic effects by affecting various ion channels (42). In one study, Somova et al. (42) showed that two triterpenoids, namely oleanolic acid and ursolic acid, isolated from *O. europaea* leaves, can behave as β-adrenergic antagonists in three models of CaCl_2_, adrenaline, and I/R—induced arrhythmias. This is similar to that seen in class IIa, where the non-selective β- and selective β1-adrenergic receptor blockers inhibit adrenergically induced G_*s*_ protein-mediated effects of increased adenylyl kinase activity, thereby suppressing SAN pacing rate (7).

#### Class (III): K^+^ channel blockers and openers

In cardiac tissue, K^+^ channels include inwardly rectifying K^+^ channels (Kir), transient outward K^+^ channels, delayed outward rectifying K^+^ channels, Ca^2+^-activated K^+^ channels, and two-pore domain K^+^ channels. They are primarily responsible for maintaining the resting potential, regulating electrical excitation, forming the peak of the AP, and repolarizing the cell (43). Any dysfunction in these channels may result in cardiac arrhythmia. Class III of AADs, including K^+^ channel blockers and openers, suppress arrhythmias by influencing AP phase 3 repolarization and ERP. This class is further subdivided into three subclasses comprising voltage-gated K^+^ (K_*v*_) channel blockers (IIIa), metabolically dependent K^+^ channel openers (IIIb), and transmitter dependent K^+^ channel blockers (IIIc). Below are several plants or their active ingredients that have been found to have antiarrhythmic efficacy similar to this class of AADs in experimental studies (7).

*Hypericum attenuatum* Fisch. ex Choisy, commonly known as St. John’s wort, has been shown to have the potential to combat cardiac arrhythmias. In one study, the rats were treated with total flavonoids extracted from *H. attenuatum* (100 mg.kg^–1^.day^–1^) for 7 days, and then the I/R model was carried out to induce arrhythmias. They also evaluated the cellular mechanism behind the probable cardioprotective effects of this extract. Results showed that the flavonoid extract could prevent I/R-induced arrhythmias through two mechanisms: (1) increasing the expression of the ATP-sensitive K^+^ (K_*ATP*_) channels subunit Kir 6.1, leading to increase their open probability, similar to the action seen in subclass IIIb of AADs, (2) reducing the expression of CaL-α1C, the most important subunit of L-type Ca^2+^ channels, which in turn leads to inhibiting the Ca^+2^ entry, similar to the class IV of AADs (44).

*Rhodiola crenulata* (Hook.f. and Thomson) H. Ohba, a Tibetan medicinal plant, has been found to be effective in suppressing cardiac arrhythmias (45, 46). In one study, Liu et al. (46) evaluated the antiarrhythmic effects of this plant in a rabbit model of heart failure (HF). The APD and ERP were found to be greater in the HF group than in the control group once AF was induced in both groups. This finding is consistent with previous evidence indicating that anatomical and structural remodeling in HF causes an increase in the ERP and that such an electrophysiological change is linked to the EAD- and DAD-induced triggered activity, thereby possibly initiating AF (47–49) found that animals treated with *R. crenulata* (270 mg.kg^–1^.day^–1^ orally) for 2 weeks had lower AF inducibility and shorter ERP and AP duration than the HF group, which received no treatment. Molecular analysis by this study showed the increased mRNA expression of K_*v*_1.5, contributing to ultra-rapid rectifier K^+^ current (*I*_*Kur*_) following treatment with *R. crenulata*, an action which may be likened to AADs with K^+^ channel activating effects. Moreover, *R. crenulata* increased the expression of Ca^2+^ -ATPase pump which restores the reduced Ca^+2^ concentration in sarcoplasmic reticulum in HF model, that might be likened to the action of subclass IVc of AADs, known Ca^2+^ channel activators Liu et al. (46).

Acacetin is a natural flavone extracted from *Saussurea tridactyla* Sch.Bip. ex Hook.f. Intraduodenal administration of this compound at doses of 2.5, 5, and 10 mg.kg^–1^ could remarkedly prevent AF in dogs through atrial selectively inhibition of *I*_*Kur*_, *I*_*to*_ and acetylcholine-activated K^+^ current (*I*_*KACh*_), represented by atrial ERP prolongation and no alteration in the corrected QT interval (50).

Berberine is a natural alkaloid found in some plants, such as *Berberis* spp. and *Coptis chinensis* Franch., shown to have the potential to suppress cardiac arrhythmias in several experimental studies (51, 52). The antiarrhythmic effects of berberine on the streptozotocin-induced model of type 2 diabetes mellitus (T2DM) in ischemic conditions have been proved by Wang and colleagues (53). They tested the antiarrhythmic effects of berberine (180 mg.kg^–1^ intragastrically for 2 weeks) in four groups: group 1: normal control rats; group 2: normal rats with myocardial infarction (MI); group 3: T2DM rats associated with MI; and group 4: T2DM rats associated with MI treated with Berberine. Berberine significantly restored the protein expression of Kir 2.1, which is impaired in diabetic and ischemic conditions, compared to untreated groups, leading to a shortening of AP duration and subsequently preventing the triggered activity in ischemic tissues. This effect can be likened to the action of subclass IIIb AADs, specifically inward rectifier K^+^ current (*I_*K*1_*) agonists, which speeds up the late phase 3 AP repolarization and stabilizes phase 4 diastolic resting potentials (7). Some mechanistic studies further studied the pharmacological targets and electrophysiological effects of berberine on isolated cardiac myocytes and found its inhibitory effects on *I_*K*1_*, slow delayed rectifier K^+^ current (*I*_*Ks*_), and *I*_*Kr*_, which subsequently could prolong the repolarization phase of AP duration and ERP, similar to the action of subclass IIIa AADs (54–56). However, these studies have been designed in non-pathologic conditions, so further studies are needed to assess these mechanisms alongside the outcomes related to arrhythmias.

Oxymatrine and its metabolites like matrine are isoquinoline alkaloids found in *Sophora flavescens* root, shown to be effective in suppressing arrhythmias (22, 57). Yong-Gang et al. (57) evaluated the effects of oxymatrine on coronary ligation-induced arrhythmias in Wistar rats. They discovered that intravenous (IV) injection of oxymatrine (10 and 20 mg.kg^–1^) prior to occlusion could decrease the VT duration, delay the onset of VT, and reduce the severity score of arrhythmias. It was shown that such protective effects are mediated by inhibition of *I_*K*1_*, reduction of *I*_*Ca–L*_, and enhancement of *I*_*to*_. The *I_*K*1_* inhibition by this phytochemical is likened to the action of subclass IIIa AADs; However, the AP duration was shortened following oxymatrine injection, and this is contrary to what is expected from subclass IIIa AADs and may be due to simultaneous effects on *I*_*Ca–L*_ and *I*_*to*_. Another study by Li et al. (51) explored the antiarrhythmic effects of matrine in a rat model of MI and confirmed the results obtained in the Gang et al. study. They found that long-term oral administration of matrine could reduce the rate of ligation-induced arrhythmias and mortality through restoring *I*_*to*_, *I_*K*1_*, and *I*_*Ca*_ impaired by MI induction, represented electrophysiologically by shortening the AP duration.

Sasanquasaponin is a triterpenoid isolated from *Camellia oleifera* Abel seeds, and its antiarrhythmic activity has been scarcely evaluated. Lai et al. (58) designed one study under both *in vitro* and *in vivo* conditions and showed that IV injection of sasanquasaponin (0.1, 0.2, and 0.4 mg.kg^–1^), 10 min prior to ischemia significantly reduced the incidence of I/R-induced VT and VF, mediated by reducing AP duration, probably through the activation of K_*ATP*_ channels, similar to that seen in subclass IIIb AADs.

Tanshinone IIA is a diterpene constituent isolated from the roots and rhizomes of *Salvia miltiorrhiza* Bunge and found to be effective in suppressing arrhythmias. Shan et al. (59) found that pretreatment of rats with tanshinone IIA (10 mg.kg^–1^.day^–1^) for 7 days before left anterior descending artery ligation could significantly reduce the duration, incidence, and severity score of arrhythmias such as VT and VF in a rat model of MI. In this study, tanshinone IIA significantly restored the protein expression of Kir2.1, which was impaired due to the overexpression of microRNA-1 during MI. Tanshinone IIA also improved *I_*K*1_* which may be as a result of increased Kir 2.1 expression. Increased *I_*K*1_* could preserve resting membrane potential in rats that received tanshinone IIA, which is probably the main underlying mechanism for protecting against ventricular arrhythmias (59). Moreover, previous evidence shows that the increased *I_*K*1_* could decrease AP duration, which subsequently prevents the EAD- and DAD-induced triggered activity in ischemic tissues. Thus, tanshinone IIA seems to exert its antiarrhythmic activity in ischemic conditions by increasing *I_*K*1_*, resulting in preserving resting membrane potential and, to some extent, preventing prolongation of AP duration and subsequent triggered activity. Such effects are similar to those exerted by *I_*K*1_* agonists in subclass III b AADs (7).

Allicin is an organosulfur compound with multiple cardiometabolic effects, isolated from *Allium sativum* L. An experimental study by Huang et al. (60) found that 28-day pretreatment with allicin could delay the onset time of BaCl_2_-induced arrhythmias and decrease the arrhythmia score. Their results showed that such beneficial effects are produced by reducing AP duration through two main mechanisms, including enhancement of *I_*K*1_* and inhibition of *I*_*Ca–L*_. As previously explained, the former mechanism is likened to the action of *I_*K*1_* agonists in subclass III b AADs (7). One other mechanistic study found the inhibitory effects of allicin on *I*_*to*_ in isolated ventricular myocytes similar to the action of subclass IIIa; however, this study has not evaluated the arrhythmia outcomes (61), and this issue should be clarified in future studies.

We discussed the antiarrhythmic effects of some active ingredients, including liriodenine, curcumin, cinnamophilin, and resveratrol, through inhibiting Nav channels in previous subsections. Studies have shown that these phytochemicals also behave like class III AADs, as they can modulate the K^+^ channel currents and affect AP duration, thereby protecting the heart against I/R-induced arrhythmias (24, 26, 29, 32, 33, 36). Note that the inhibition of Ca^2+^ channels by curcumin, cinnamophilin, and resveratrol might alleviate the prolonged AP duration exerted by inhibiting K^+^ channels.

The antiarrhythmic effects of WXKL through inhibiting Na^+^ current were discussed in previous subsections. One study looked at the other mechanisms of antiarrhythmic action of WXKL and showed that 3-week administration of WXKL (8 g.kg^–1^.day^–1^) before ischemia induction could significantly reduce the incidence and number of VT, VF, and ventricular ectopic beats in a rat model of I/R-induced injury *via* inhibiting the *I*_*Ca–L*_ and *I*_*to*_ (62). The latter is similar to the action subclass IIIa AADs.

As we mentioned above, the SSYX capsule, as a mixture formulation of herbal drugs, has been traditionally used for treating cardiac arrhythmias in China by targeting multiple ion channels in the heart (16). In one study, Zhao et al. (63) revealed that pretreatment of rats with SSYX capsule (1.8 g.kg^–1^.day^–1^) for 7 days could significantly reduce the incidence of VT, VF, and arrhythmias severity scores and delay the initiation of coronary occlusion-induced arrhythmias. In this study, SSYX significantly prolonged AP and slowed cardiac repolarization by inhibiting *I_*K*1_* and *I*_*to*_, which subsequently changed the myocardial refractoriness and conductivity and exerted a therapeutic effect on ischemic arrhythmias. Such an effect is similar to that exerted by subclass IIIa, which has been shown to be effective in combating reentry-induced cardiac arrhythmias.

#### Class (IV): Ca^2+^ handling modulators

The main Ca^2+^ channels in cardiac cells are transmembrane L-type and T-type, as well as intracellular channels. Calcium channels in cardiac cells are responsible for regulating cardiac pacemaking, AV conduction, and heart rate determination (64). Ca^2+^ handling modulators are known as Class IV antiarrhythmics. According to modernized classification, this class of AADs is divided into five subclasses (IVa–IVe), the first two of which, surface membrane Ca^2+^ channel blockers (IVa) and intracellular Ca^2+^ channel blockers (IVb), have been clinically approved for the treatment of certain types of cardiac arrhythmias. The agents in the IVa subclass are the most commonly used drugs. They are primarily used to treat supraventricular arrhythmias caused by reentry or triggered activity by reducing AV nodal conduction (7).

*Panax notoginseng* (Burkill) F. H. Chen is a Chinese medicinal plant that contains multiple saponins shown to be protective against CVDs (65). Guan et al. (66) tested the antiarrhythmic effects and mechanism of action of ginsenoside-Rb_1_, a component of *P. notoginseng* saponins, through *in vivo* and *in vitro* investigations. They found that Rb_1_ (30 and 50 mg.Kg^–1^) reduced BaCl_2_-induced VTs in the rat model and CaCl_2_-ACh-induced AF and chloroform-induced VF in the mouse model. *In vitro* study on heart cells showed that Rb_1_ restored the isoprenaline-induced increased intracellular Ca^2+^ concentration by inhibiting its entry through Ca^2+^ channels in a concentration-dependent manner (66).

As mentioned in the previous subsections, WXKL, allicin, and total flavonoid from *Hypericum perforatum* have protective effects against arrhythmias induced by the I/R model or BaCl_2_ injection. Regarding WXKL, *in vitro* investigations of isolated ventricular myocytes showed that one other mechanism responsible for such beneficial effects is the inhibition of *I*_*Ca–L*_ (62) similar to that exerted by L-type voltage-gated Ca^2+^ (Ca_*v*_) channel blockers in subclass IVa AADs, such as verapamil and diltiazem (7). Regarding Allicin, as we earlier stated, Huang et al. (60) study proved its antiarrhythmic effects associated with shortening of AP duration, mechanistically caused by *I*_*Ca–L*_ inhibition, resemble the action of subclass IVa AADs. In the case of total flavonoid from *H. attenuatum*, Ma et al. (44) found that 7-day pretreatment of rats with this extract could reduce I/R-induced arrhythmias *via* a mechanism similar to the action of class III and class IV AADs.

Flavonoid extract from *Viscum coloratum* (Kom.) Nakai is thought to have cardioprotective effects. Wen-Feng et al. (67) investigated the antiarrhythmic effect of *V. coloratum* on aconitine-induced arrhythmias in rats. They found that pretreatment of rats with an IV injection of *V. coloratum* could significantly alleviate the susceptibility to premature ventricular contraction (PVC), VT, and VF. In this study, *V. coloratum* significantly produced an inhibitory effect on *I*_*Ca–L*_ and shortened the AP duration of ventricular myocytes, similar to that seen in subclass IVa AADs.

As discussed in previous subsections, oxymatrine and its metabolites like matrine found in *S. flavescens* root have a protective role against ischemia-induced and aconitine-induced arrhythmias. Evidence shows that these alkaloids, in addition to their inhibitory action on Na^+^ and K^+^ channels, can also block the *I*_*Ca–L*_, resembling the class IV of AADs (22, 57).

We discussed in previous subsections that some phytochemicals, including curcumin, cinnamophilin, barbaloin, and resveratrol, exert their antiarrhythmic effects through modulating multiple ion channels. One of the mechanisms behind their beneficial effects involves blocking the *I*_*Ca–L*_, similar to the action of class IV AADs (26, 27, 29, 30, 32, 33, 36). As mentioned above, inhibiting *I*_*Ca–L*_ in such agents with multiple actions can moderate the AP prolongation that occurs by concurrent inhibition of K^+^ and Na^+^ channels, which could be considered as a starting point for developing future AADs without serious side effects like QT prolongation. Also, as we stated above, *Rhodiola crenulata* has protective effects against arrhythmias through affecting the expression of multiple ion channels, among which the increased expression of sarcoplasmic reticulum Ca^2+^ ATPase is likened to the action of subclass IVc of AADs (46).

#### Class (V): Mechanosensitive channel blockers

Transient receptor potential channels are mechanosensitive ion channels that have several subfamilies, among which TRPC1, TRPC3, and TRPC6 are related to cardiac function and are involved in intracellular Ca^2+^ signaling and the regulation of heart function. They are closely linked to the development of cardiac hypertrophy, fibrosis, arrhythmias, and myocardial infarction (68, 69). Inhibition of such channels has recently been considered to be a promising therapeutic target for treating some types of cardiac arrhythmias by reducing EAD-/DAD-induced TAs, known as Class (V) AADs according to modernized classification (7).

In the case of this class of AADs, our review did not find any study that concurrently evaluated the antiarrhythmic effects and such a mechanism of action of herbal medicine. However, we found a small numbers of plant extracts and multi-component herbal preparations, as described below and summarized in Table 2, whose inhibitory effects on mechanosensitive ion channels have been identified in *in vitro* studies, while their antiarrhythmic effects have been evaluated in some other separate studies. For instance, *S. miltiorrhiza*, known as Danshen in China, is one of the main components of a Chinese multi-component herbal preparation, namely Danqi soft capsule (DQ), found to be effective in suppressing cardiac arrhythmias (70). Salvianolic acid B, a bioactive compound found in the roots of *S. miltiorrhiza*, has been shown to be capable of inhibiting TRPC3 and TRPC6 mediated Ca^2+^ overload in rat cardiomyocytes (71). Thus, the antiarrhythmic effects of *S. miltiorrhiza* might be, at least in part, due to the inhibitory effects of Salvianolic acid B on TRPC channels. However, further studies are warranted to concurrently evaluate the efficacy and mechanism of action of this medicinal plant.

#### Class (VI): Gap junction channel modulators

Gap junctions are intercellular junctions composed of different subunits, namely connexin (Cx) proteins. Three major connexin isotypes are expressed in the heart: Cx43, Cx45, and Cx40. Connexins play an essential role in electrical coupling by modulating intercellular currents; they also influence AP conduction, cardiomyocyte death, and survival (72). The instability of gap junctions in response to pathophysiological signals, either by disrupting or over-expressing their subunits, can increase susceptibility to cardiac arrhythmias (73). Several agents acting as Cx openers or blockers have shown promising results in suppressing cardiac arrhythmias, known as Class VI in the modernized classification of AADs (7). Below, we summarized the results of experimental studies that investigated the antiarrhythmic effects of herbal medicine and concurrently determined the mechanism of antiarrhythmic action.

*Nardostachys chinensis* Batalin is a TCM shown to have potential cardioprotective effects in some studies (74, 75). One study by Zhang et al. (75) evaluated the protective effects of *N. chinensis* against spontaneous ventricular arrhythmias in a rat model of hyper-acute MI. Their results showed that pretreatment of rats with *N. chinensis* rhizome extract (600 mg.kg^–1^.day^–1^) for 30 days could reduce the incidence of VF, VT, and PVCs induced by I/R injury (75). The molecular analysis of ventricular myocyte tissues showed a reduced degradation rate of Cx43 in the treatment group, which might be the possible mechanism underlying the observed antiarrhythmic effects.

Grape seed proanthocyanidin extract (GSPE), a polyphenol derived from grape seeds, has been found to protect against cardiac arrhythmias (76, 77). Liang et al. (77) found that pretreatment of rabbits with GSPE (100 or 200 mg.kg^–1^) for 21 days significantly lowered the incidence of I/R-induced VF. The immunohistochemistry results showed that the expression of Cx43, impaired by I/R injury, was significantly improved in the GSPE group, which may provide a mechanism for antiarrhythmic effects similar to the effect of Gap junction channel modulators in class VI of AADs. Another study on a rat model of I/R injury showed that GSPE could reduce the incidence of I/R-induced ventricular arrhythmias through increasing the expression of Na^+^/K^+^-ATPase α_1_-subunit, confirmed by western blot analysis (76). Evidence shows that Na^+^/K^+^-ATPase α_1_ activity is necessary for the formation of gap junction proteins (78) and can modulate the cardiac remodeling that occurs in the failing heart (79). Hence, the increased expression of Cx43 and improving gap junctional communication observed in the Liang et al. study might be, at least in part, due to the increase in Na^+^/K^+^-ATPase activity by GSPE administration.

Linalool is a natural monoterpene extracted from aromatic plant-derived essential oils. Cardioprotective effects of Linalool against I/R injury have been found in several experimental studies (80, 81). Ke et al. (81) found that pretreatment of rats with linalool (50 or 100 mg.kg^–1^.day^–1^) for 7 days before LAD occlusion significantly decreased the prevalence, duration, and severity score of ventricular arrhythmias. Through molecular analysis, they proved the preventive effect of linalool against Cx43 degradation occurred by ischemic injury, which may be an underlying mechanism for the observed antiarrhythmic effects.

Tongguan capsule is a patented TCM consisting of four natural compounds, shown to be effective in CVDs (82). The antiarrhythmic effect of the Tongguan capsule has been proven only by one experimental study. Ma et al. (83) found that 4-week treatment with Tongguan capsule could significantly reduce susceptibility to arrhythmias in the post-MI rat model, probably through two main underlying mechanisms: (1) increasing the expression of Cx43, (2) reducing interstitial fibrosis, hypertrophy and left ventricular remodeling. These mechanisms are similar to those seen in classes VI and VII of AADs, respectively.

Danqi soft capsule is a combination of *S. miltiorrhiza* and *P. notoginseng*, two herbal medicines with cardioprotective effects (70). Ma et al. (70) showed the antiarrhythmic effect of low, middle, and high doses of DQ on MI-induced arrhythmia in rats. They found that all doses of DQ could significantly decrease cardiac remodeling, VT score and VT inducibility. They also found that DQ could reverse the downregulation of Cx43 and inhibit cardiac fibrosis formation *via* inhibiting the transforming growth factor-beta1 (TGF-β1)/Smad3 pathway. These are similar to those seen in class VI and VII of AADs that decrease arrhythmia by modulating gap junctions and preventing cardiac remodeling and hypertrophy, respectively.

Fumai granule is a Shengmai San-derived TCM containing a mixture of six medicinal herbs that have protective effects against arrhythmias. One study by Ma et al. (84) has tested the efficacy of this multi-component herbal preparation in preventing AF in a rat model of ischemic HF. They found that Fumai granule (500 mg.kg^–1^.day^–1^) for 4 weeks following the induction of ischemic HF could significantly reduce the inducibility and duration of electrical stimulation-induced AF. They also described two mechanisms for such effects, which include: (1) increasing the expression of Cx43 and Cx40 to resemble the action of class VI AADs, and (2) inhibiting cardiac fibrosis and inflammation through affecting the TGF-β1 pathways and reducing inflammatory markers such as interleukin-6 (IL-6), monocyte chemoattractant protein-1, tumor necrosis factor-α, and B-type natriuretic peptide.

#### Class (VII): Upstream target modulators

Evidence shows that inflammation and myocardial fibrosis can increase the risk of developing arrhythmias (85, 86). Several anti-fibrotic and anti-inflammatory agents were shown to have antiarrhythmic action through limiting pro-arrhythmia substrates such as post-infarct myocardial fibrosis and remodeling (87, 88). These drugs mostly have other cardioprotective effects, and though their antiarrhythmic efficacy has not been approved yet, they might be considered as potential therapeutic choices (88). Such cardioprotective non-antiarrhythmic drugs with potential antiarrhythmic action are classified as upstream target modulators in the modernized AADs classification (7). Below we described medicinal herbs or their active constituents with antiarrhythmic action like upstream target modulators.

*Arnebia euchroma* (Royle) I. M. Johnst. Radix and its active ingredients have been shown to be effective against CVDs (89, 90). Zhou et al. (89) have investigated the antiarrhythmic effects of this plant and found that 1-week pretreatment of rats with *A. euchroma* Radix (0.18 g.mL^–1^) could significantly reduce the incidence and duration of ACh-CaCl_2_-induced AF similar to that exerted by amiodarone as the positive control. Through an *in vitro* assay, *A. euchroma* Radix diminished atrial fibrosis and enlargement induced by ACh-CaCl2, which suggests that this mechanism may be responsible for the anti-AF effects exerted by this plant (89). Such a mechanism is similar to the action of upstream target modulators in the class VII AADs.

The antiarrhythmic effects of *R. crenulata* and its ability to act like class III and class IV of AADs were stated in previous subsections. Another mechanism proposed for the antiarrhythmic effects of *R. crenulata* is the regulation of inflammatory cellular signaling pathways. Hsiao et al. (45) found that *R. crenulata* and its main active ingredient, salidroside, exerted their antiarrhythmics effects by reducing the expression of inflammatory markers and inhibiting fibrosis, collagen genesis, and cellular pathways leading to apoptosis.

Hesperidin is a bioflavonoid mainly found in medicinal plants belonging to the *Citrus* spp. Gandhi et al. (91) investigated the antiarrhythmic effects of hesperidin (100 mg.kg^–1^) for 15 days in an *in vivo* model of I/R myocardial injury and found a considerable reduction in the incidence and duration of VT and VF and the severity score of the arrhythmias. Hesperidin significantly increased the endogenous antioxidant enzymes such as SOD and CAT, decreased oxidative stress and infarct size, and also exhibited anti-inflammatory action. These effects are likened to the action of upstream target modulators. Similarly, Pashai et al. (92) confirmed the protective effects of Hesperidin against I/R-induced arrhythmias and found that such effects are mediated by activating signaling pathways, leading to the inhibition of proinflammatory conditions.

Troxerutin is a flavonol-type flavonoid isolated from *Styphnolobium japonicum* (L.) Schott and *Dimorphandra gardneriana* Tul., and has been studied for its antiarrhythmic effect in one study. Najafi et al. (93) investigated the effect of troxerutin on I/R-induced arrhythmia in diabetic and healthy rat-isolated hearts. They showed that pretreatment of rats with troxerutin (150 mg.kg^–1^.day^–1^) for 4 weeks decreased the duration and incidence of PVC, VT, and suppressed VF. Moreover, troxerutin decreased the arrhythmias severity score in diabetic rats more than the control group. They further evaluated the mechanism behind such antiarrhythmic effects and found that troxerutin could remarkably reduce pro-inflammatory cytokines, similar to that described for upstream target modulators (93).

Magnolol, a lignan compound isolated from *Magnolia officinalis* Rehder and E. H. Wilson, has been found to be effective in preventing and suppressing cardiac arrhythmias (94, 95). Lee et al. (95) surveyed the effect of magnolol on I/R-induced arrhythmias in rats and concurrently evaluated its mechanism of action. They showed that pretreatment of rats with magnolol (0.2 and 0.5 μg.kg^–1^) before coronary ligation significantly abolished the VF development and reduced the duration of VT and VF. Through *in vitro* investigation, magnolol showed anti-inflammatory effects, indicated by reduced neutrophil migration in ischemic myocardium, which may explain its antiarrhythmic effects.

As we described in previous subsections, saponin compounds found in *P. notoginseng* have antiarrhythmic effects. In one study by Li et al. (96), these saponins were found to be effective in reducing the duration of CaCl_2_-ACh mixture-induced AF when administrated 1 h before model induction at the doses of 100 and 150 mg.kg^–1^ daily for 7 days. In this study, further insights into the mechanism showed the antioxidant and anti-inflammatory properties of this compound, which may explain, at least in part, its antiarrhythmic effects.

Anthocyanins are colored pigments of some flavonoid compounds found in several plants, such as bilberry (*Vaccinium myrtillus* L.) (97). Ziberna et al. (97) studied the antiarrhythmic effect of bilberry anthocyanins on I/R induced arrhythmia in rats isolated hearts. Their study showed that the perfusion of hearts with low concentration of bilberry anthocyanins (0.1, 1, 5, and 10 mg.L^–1)^ could significantly reduce the incidence and duration of arrhythmias such as VF. The mechanism behind the antiarrhythmic effects of anthocyanins was found to be its antioxidant and anti-inflammatory properties, similar to the action of upstream target modulators.

Arctigenin is a lignan derived from *A. lappa*, shown to have anti-inflammatory and cardioprotective effects (28, 98). Yang et al. (28) have evaluated the antiarrhythmic and antioxidant effects of ATG in a rat model of I/R injury. They showed that pretreatment with ATG (50 mg.kg^–1^.day^–1^ and 200 mg.kg^–1^.day^–1^) could significantly prevent ventricular arrhythmias and reduce their duration during ischemia and reperfusion phases. Molecular analysis showed that ATG could remarkably increase the expression of nuclear factor erythroid 2-related factor 2 (Nrf2), a transcription factor modulating the cellular response to oxidative stress and inflammation, and some other antioxidant molecules. Considering the key role of activating Nrf2 signaling pathways in suppressing inflammatory processes (99), increasing the expression of Nrf2 following ATG administration seems to be an underlying mechanism for protecting the myocardium from cellular injuries and subsequent arrhythmias.

As described in the previous subsections, SSYX, DQ, and Fumai granule are multi-component herbal preparations demonstrated to be useful antiarrhythmic agents in experimental studies. One study by Ma et al. (16) showed that 4-week administration of SSYX (600 mg.kg^–1^.day^–1^) could significantly reduce the duration and vulnerability of programmed electrical stimulation-induced AF in a rat model of MI. This study also found that such effects might occur by inhibiting the expression of inflammatory factors and reducing fibrosis, similar to the effects exerted by upstream target modulators. Regarding the antiarrhythmic effects of the DQ capsule, Ma et al. (70) found the protective effects against MI-induced arrhythmia *via* inhibiting cardiac fibrosis formation in the rat model of MI, similar to the action of upstream target modulators. In the case of Fumai granule, the protective effects against electrical stimulation-induced AF were thought to be mainly related to the inhibition of cardiac fibrosis and inflammatory statues (84).

Ping-Lv-Mixture (PLM) is a Chinese multi-component herbal preparation consisting of a mixture of five medicinal plants. An and Yang found that 7-day pretreatment of rats with PLM (0.04, 0.2, and 1 g.kg^–1^.day^–1^) could significantly reduce the incidence and duration of VT, VF, and PVCs in the rat model of myocardial I/R injury (100). This study also showed that such protective effects might be mediated by antioxidative and anti-inflammatory mechanisms, assembling the action of upstream target modulators.

Yindanxinnaotong capsule is a Chinese patented multi-component herbal preparation, shown to have antiarrhythmic effects through affecting inflammatory pathways. One study by Wang et al. (101) found that the types of Yindanxinnaotong formulations which contain *G. biloba* and *S. miltiorrhiza* extracts could reduce the rate of I/R-induced ventricular arrhythmias in a rat model. *In vitro* investigation showed a reduction in myocardial injury markers and inflammatory factors, which may be responsible for the observed antiarrhythmic effects.

#### Clinical trial studies

Here we reviewed the available clinical studies which evaluate the efficacy and safety of multi-component herbal preparations and bioactive compounds for the treatment of cardiac arrhythmias in humans. The results are summarized in Table 4.

##### Dingji Fumai decoction

Dingji Fumai Decoction is a mixture of several herbal medicines developed to treat palpitations (39). Liang et al. (39) conducted a double-blind, randomized, placebo-controlled trial including 92 patients to investigate the safety and efficacy of adding DFD to metoprolol for treating PVCs and compare the results with the metoprolol alone group as the positive control group. The number of PVCs was significantly reduced in DFD combined with the metoprolol group compared to the metoprolol alone group. Moreover, no side effects were reported in the DFD treatment group. These researchers performed another double-blinded, randomized, placebo-controlled trial to investigate the impact of DFD on PVC in a larger population (102). In this study, 136 patients (71 in the DFD group and 65 in the control group) completed the study. The results revealed that after 12 weeks of treatment with DFD or placebo, the number of PVCs and the severity of palpitations remarkably decreased in the DFD arm. Also, patients had good compliance with the treatment, and DFD was well tolerated with no adverse drug reactions in the DFD arm.

##### Shenmai injection

Shenmai is a traditional Chinese herbal mixture mainly consisting of *Panax ginseng* C. A. Mey. and *Ophiopogon japonicas* (Thunb.) Ker Gawl. Shenmai injection is a Chinese patent injection that has been effective as adjunctive therapy for coronary heart disease. An open-label, non-randomized controlled trial by Deng et al. (103) studied the efficacy and safety of shenmai injection combined with amiodarone in suppressing paroxysmal AF. A total of 351 patients were enrolled in the study: 223 patients in the treatment group (shenmai injection + amiodarone) and 128 patients in the control group (amiodarone alone). The results of this study showed that the total efficacy rate had no significant difference between the two groups; however, in the treatment group, the ventricular rate was significantly lower than in the control group. Also, in the treatment group, the cardioversion rate was considerably higher, and it took less time to reach normal rhythm compared to the control group. Patients in both groups only experienced mild adverse drug reactions and there were no further adverse effects.

##### Baicalin

Baicalin is a flavone-type flavonoid isolated from the dried root of *Scutellaria baicalensis Georgi*. Xiao et al. (104), in an open-label, randomized controlled trial, investigated the efficacy of baicalin in patients with aconitine toxicity. A total of 60 patients were included in the study, divided into the treatment group (baicalin + conventional therapy) and the control group (conventional treatment alone). The study showed that patients in the baicalin group had much faster relief from symptoms of premature beats, auricular flutter, and auricular fibrillation and had a shorter recovery time compared to the control group.

##### Shensong Yangxin

Shensong Yangxin capsule is an approved herbal medicine for treating arrhythmias in China (105). Wang et al. (105) investigated the efficacy and safety of the SSYX capsule in treating PVCs in a double-blinded, randomized, multicenter clinical trial in 411 patients. Results showed that 12-week treatment with SSYX remarkably reduced the total number of PVCs and improved left ventricular ejection fraction compared to the placebo group. In another study, Zou et al. (106) conducted a double-blinded, placebo-controlled multicenter randomized clinical trial to evaluate the efficacy and safety of SSYX capsules in the treatment of PVCs in 677 patients with organic and 188 patients with non-organic heart diseases. They found that 8-week therapy with SSYX capsules significantly decreased the number of PVCs compared to the control group. Only one patient in the non-organic group and four in the organic group reported mild adverse effects with SSYX, which all disappeared after a few days.

##### Wenxin Keli

Wenxin Keli, a mixture of herbal medicines, as earlier described, has been reported to have therapeutic effects on cardiac arrhythmias (37). Hua et al. (107) conducted a randomized, double-blind, controlled clinical trial to determine the efficacy and safety of WXKL in treating patients with frequent PVCs. A total of 1071 patients completed the study (536 cases in the treatment arm and 535 cases in the placebo arm). The results of the study showed that a 4-week treatment with WXKL (9 g three times daily) significantly lowered the number of PVCs and their related symptoms compared to the placebo. Only three patients in the WXKL reported mild side effects, probably unrelated to the WXKL.

##### Xin Su Ning

Xin Su Ning (XSN) is a Chinese multi-component herbal preparation that has been patented for treating ventricular arrhythmia. Ma et al. (108) investigated the efficacy and safety of XSN in treating PVCs in a three-armed, double-blind, randomized controlled trial on 861 patients. A total of 779 patients completed the study, and results revealed that a 4-week treatment with XSN or mexiletine significantly lowered the number of PVCs and their related symptoms. Xin Su Ning had a higher rate of significantly effective responses than the placebo. Only mild side effects were reported, which probably were not related to the XSN. This study also reported the results of an animal study conducted to investigate the antiarrhythmic mechanism of this multi-herbal component. They found that XSN dose-dependently inhibited the human Na_*v*_1.5 channel at its inactivation state, suggesting a post-repolarization refractoriness period, which provides a mechanistic explanation for the XSN effect on the premature heart beat in patients. Xin Su Ning also inhibited the hERG K^+^ channel, which subsequently prolonged APD and increased the ERP of the heart, leading to PVC inhibition.

## Discussion

This review targeted the antiarrhythmic effects and their underlying mechanisms of plant extracts, phytochemicals, and multi-component herbal preparations in preclinical and clinical studies. Below, we summarize the important results from reviewed studies and discuss the information gaps and research priorities.

In the present study, three plants extracts, 26 phytochemicals, and eight multi-component herbal preparations were found to be encouraging compounds for developing new antiarrhythmic drugs. The phytochemicals reviewed in this study were mostly polyphenol compounds (flavonoids and non-flavonoids), alkaloids, and terpenoids. The multi-component herbal preparations consisted of several herbal medicines; some were studied individually for their antiarrhythmic effects. Four multi-component herbal preparations were patented for their antiarrhythmic and cardioprotective effects.

The reviewed plants, multi-component herbal preparations, and phytochemicals had different electrophysiological properties and mechanisms of action, as shown in Figures 3, 4. Most of them exhibited their antiarrhythmic effects by activation or inhibition of ionic current or by affecting the expression of the cardiac ion channels. The modulation of gap junction proteins (Cx43 and Cx40), and elimination of the arrhythmia’s substrate, as upstream targets (e.g., fibrosis, remodeling, and inflammation) were the other investigated antiarrhythmic mechanisms. In some reviewed studies, the exact mechanism of action was not investigated, and the authors discussed some therapeutic mechanism hypotheses based on previous studies’ results. Although most plants demonstrated anti-oxidant properties, only some of them were clinically relevant for controlling cardiac arrhythmias. Also, the lack of information on cellular electrophysiological effects is another gap in reviewed studies. Understanding these information gaps will certainly develop the application of medicinal plants to prevent and treat diseases. Thus, further work to identify the therapeutic mechanisms and electrophysiological effects of plants, in parallel with exploring their pharmacological activity, is needed.

Most of the reviewed studies did not determine the phytochemical composition of the medicinal plants and just focused on previous studies to justify their pharmacological claims. The concurrent study of phytochemicals and mechanism of action should be investigated further to determine the main chemical compounds responsible for therapeutic effects. Also, some studies did not introduce the origin of medicinal plants, influencing the active constituents probably due to the various environmental factors. Many studies have proved that environmental differences, such as climate, soil parameters, and altitude, in different places of origin influence the content of active ingredients and, therefore, the pharmacological activity of medicinal plants (109). Thus, this issue should be a matter of interest in future research.

There are also some other information gaps in the method of the reviewed studies that need to be explored in future studies. Based on our quality assessment, as shown in Figure 2, the most detected biases were attributed to blinding and randomization, which are given little attention in animal studies. Also, the majority of studies had a short duration, which might weaken the level of evidence. Moreover, a high level of uncertainty in these studies, including inadequate information on optimal dose, the minimal active concentration, the frequency of administration, toxicity evaluation, the methods of specimen collection, sampling, and statistical analysis, may lead to reduced reproducibility and replicability, according to “Guidelines for submission in peer-reviewed pharmacological literature” (110).

This review emphasizes the importance of research on the cardioprotective of medicinal plants and their bioactive compounds to provide guidance for the future development of new antiarrhythmic drugs. The question is, what approach can bridge the gap between herbal medicine and modern pharmacology, and how can medicinal plants be viewed through the lens of modern pharmacology? Medicinal plants typically contain hundreds of active compounds, the identification of which is essential for comprehending the mechanism of the entire plant. Although discovering an active compound and describing its effects on pharmacological targets is useful in identifying lead compounds for drug discovery, investigating the effects of multiple compounds together and their synergistic effects may be more valuable. Multiple active ingredients of a single plant can simultaneously act on different cellular targets and together show a beneficial cumulative effect on a specific disease that may be superior to using each isolated component separately (111, 112). In other words, these compounds can potentially act as a “polypill,” a concept in modern medicine that was first introduced by Wald and Low in 2003 as a single pill combining several drugs with fixed doses for the prevention of CVDs (113). Combination therapies in modern medicine may be preferred in some chronic diseases such as cardiovascular disease and cancer due to their greater efficacy, lower resistance, and higher compliance rate over monotherapy. Cardiac arrhythmias are also complex diseases that are linked to the interacting results of multiple molecular, cellular, and physiological pathways. Investigating multiple compounds which simultaneously target multiple arrhythmogenic pathways may help to identify more effective and lower toxicity multi-target AADs. For instance, one of the issues in atrial arrhythmia management relates to the use of AADs that, in addition to their beneficial effect, lengthen the AP of ventricular cells, represented by QT prolongation in the electrocardiogram, which can itself trigger hazardous ventricular arrhythmias. The solution of modern medicine is to find drugs that selectively act on the atria and have minimal effect on the ventricles. Through this review, we also found some phytochemicals and multiple-component herbal formulations, like WXKL, that have selective antiarrhythmic effects against atrial arrhythmias without having a major effect on the AP of the ventricles. Regarding WXKL, evidence shows its inhibitory effect on multiple ion channel currents (7, 38), possibly indicating that different active ingredients of such herbal formulations have targeted desirable ion channels, which have led to its acting as an atrial-selective agent. Investigating active ingredients of such herbs using scientific methods to clarify their mechanisms individually and their pooled effects and interactions when used together, and subsequently optimizing combinations in terms of dose-response, can be a starting point for developing future atrial-selective AADs with a minimum adverse effect on ventricules. This is precisely what “Network Pharmacology” does; a new paradigm in drug discovery that examines the underlying pathologic pathways of diseases as a network in order to combine different synergistic agents to create an optimal formula with the greatest efficacy and the fewest side effects for a specific disease (112).

Overall, this review tried to find plant-derived natural agents, including herbal extracts, phytochemicals, and herbal formulations which potentially could be considered as lead compounds for future drug discovery in the field of cardiac arrhythmias. Classification of agents based on the modernized classification of AADs may help researchers design comparative studies of better quality and identify the therapeutic potential of these medicinal plants for their use as a new medicine. Gaps in study design, phytochemistry, pharmacology, and toxicology information can dispraise the reproducibility of these pieces of evidence. Thus, more experimental and clinical studies of better quality should be designed to bridge these gaps and validate the relationship between their uses in traditional medicine and modern pharmacology.

## Author contributions

DS, BA, and AV-F contributed to the conception and design of the study. DS and BA contributed to search and data collection. DS, AV-F, and RR supervised the research. DS, BA, AV-F, RR, AT, and HR wrote the original draft of the manuscript. BA prepared the figures for the manuscript. All authors contributed to manuscript revision and read and approved the submitted version.

## Abbreviations

AADs: antiarrhythmic drugs
CVDs: cardiovascular disease
I_*f*_: pacemaker current
SAN: sinoatrial node
SSYX: Shensong Yangxin
AP: action potential
Nav: voltage-gated Na^+^
I_*Na*_: sodium currents
Cav: voltage-gated Ca^2+^
WXKL: Wenxin Keli
ACh: acetylcholine
AF: atrial fibrillation
ERP: effective refractory period
V_*max*_: action potential upstroke
I_*Na–L*_: late sodium channel current
I_*Na–T*_: transient sodium currents
DFD: Dingji Fumai decoction
I_*Ca*_: calcium currents
I_*Ca–L*_: L-type calcium currents
DADs: delayed afterdepolarization
I/R: ischemia-reperfusion
EC50: Half maximal effective concentration
I_*to*_: transient outward potassium current
VT: ventricular tachyarrhythmias
VF: ventricular fibrillation
EAD: early afterdepolarization
*I*_*Kr*_: rapid delayed rectifier potassium currents
I_*SS*_: sustained outward potassium currents
Kv: voltage-gated K^+^
hERG: Human Ether-a-go-go-related Gene mediated potassium currents
*I*_*K1*_: inward rectifier potassium current
T2DM: type 2 diabetes mellitus
MI: myocardial infarction
K_*ATP*_: ATP-sensitive potassium channel
Kir: Inwardly rectifying potassium channel subunit
I_*K*_: outward delayed potassium current
I_*Ks*_: slow delayed rectifier potassium current
AV: atrioventricular
PVC: premature ventricular contraction
IV: intravenous
TRPC: Transient Receptor Potential Channel
DQ: Danqi soft capsule
Cx: connexin
TGF- β 1/Smad3 pathway: transforming growth factor-beta1
TCM: traditional Chinese medicine
IL: interleukin
GSPE: Grape seed proanthocyanidin extract
PLM: Ping-Lv-Mixture
Nrf2: nuclear factor erythroid 2-related factor 2.
