# Supplementary material for: Mechanism-based targeting of cardiac arrhythmias by phytochemicals and medicinal herbs: A comprehensive review of preclinical and clinical evidence
Source: Front Cardiovasc Med. 2022 Sep 29;9:990063. doi: 10.3389/fcvm.2022.990063 (PMC9559844; doi:10.3389/fcvm.2022.990063)
Supplement: Supplementary file 1 [file Data_Sheet_1.docx]

Supplementary Material

Supplementary Table 1: Plant extracts with antiarrhythmic effects and their probable mechanism of action

| Plant extracts | | | | | | | |
| --- | --- | --- | --- | --- | --- | --- | --- |
| Scientific name of plant(s) | Type of extract and active constituents | Study design/ model | duration | Dosage/  intervention | Results | Probable Mechanisms | References |
| *Aralia elata var. mandshurica*(Rupr. & Maxim.) J.Wen | Whole plant Water extract | Wistar rats/  I/R-induced arrhythmia | 5 days | 16 mg/kg/day  Intragastric | ↓ the incidence of VT, ventricular extrasystole  ↓ Arrhythmia severity index | ↑ opioid peptides in the blood and tissues of rats | (1) |
| *Allium sativum*L. | Water extract of Bulb | Mongrel dogs of either sex/  Ouabain induced arrhythmia | Acute administration | 52.00±28.20 mg/kg and 125.00 ± 20.30 mg/kg  IV administration | Suppression of PVC and  VT induced by ouabain | Normalizing intracellular calcium | (2) |
|  |  | Male rats isolated atria/  Isoprenaline or Aconitine induced arrhythmia | 5 min | 1.35±0.21  mg/ml and 1.55±0.38 mg/ml  Perfusion | Suppression of the tachycardia induced by isoprenaline, and ectopic rhythm caused by aconitine |  |  |
|  | Bulb powder | Juvenile pigs of either  Sex/  Electrical stimulation | Acute administration | 20 and 40 mg/kg  IV injection | ↓ upper limit of vulnerability  ↑VF threshold |  | (3) |
|  | Hydro-ethanol extract of bulb | barren ewes/  *Nerium oleander* induced arrhythmia | Acute administration | 50 mg/kg  IV infusion | delayed the initiation of  arrhythmia and increased the survival rate |  | (4) |
| *Allium ursinum*L. | pulverized dried leaves | male Wistar rats isolated heart/  I/R-induced arrhythmia | 8 weeks | 2 g/100g/day  PO | ↑ the time until occurrence of ventricular tachycardia  ↓ incidence of VF and VT |  | (5) |
| *Bauhinia variegata*L. | Ethanolic root extract | Wistar rats/  CaCl_2_-induced arrhythmia | Acute administration | 400 mg/kg  IV injection | ↓incidence of AF and VF | Antioxidant | (6) |
| *Cinnamomum verum*J.Presl | Dried barks  hydroethanolic extract | Male SD rats/  I/R-induced arrhythmia | 14 days | 50, 100, and 200 mg/kg/day  Intragastric | ↓ mean number of VEB episodes  ↓ the number of VT episodes  ↓ total duration of VTs | Antioxidant | (7) |
| *Citrus medica*L. | Ethanol extract of the whole fruit | Male Wistar rats/ Isoproterenol induced arrhythmias | 15 days | 250 and 500 mg/kg/day  PO | ↓ cardiac disturbances and tachycardia | Antioxidant  Anti-inflammatory | (8) |
| *Crocus sativus*L. | Aques extract of dried stigmas | Male Wistar rats/  I/R-induced arrhythmia | 7 days | 50, 100 and 200 mg/ml/kg/day  PO | ↓ incidence and duration of VF  ↓ Duration of VT and VT/VF in the100mg extract group  ↓ score of arrhythmia severity | Antioxidant  Inhibition of Ca channels | (9) |
| *Crataegus spp* | Ethanolic extract from hawthorn leaves with flowers | Male Wistar rats/  I/R-induced arrhythmia | 7 days | 10 or 100 mg/kg/day  PO | ↓ incidence of VT, VF, and mortality | Antioxidant  Inhibition of K channels | (10) |
|  | flowering tops  hydroalcoholic, ethyl acetate, and chloroform extract of *Crataegus meyeri* | male Wistar rats/  I/R-induced arrhythmia | 30 min | 1 mg/kg/min  IV infusion | ↓ total number of  ectopic beats  ↓ reversible VF duration |  | (11) |
|  | *Methanolic* extract of leaves and flowers of *Crataegus oxycantha* | Male Wistar rats isolated heart/  I/R-induced arrhythmia | 3 month | 2g/100g/day  PO | ↓ prevalence of VT  ↓ risk of malignant arrhythmia  ↓ duration of the VT episodes |  | (12) |
|  | Dry fruits alcoholic extract of *Crataegus oxyacantha* | Male Wistar rats/  Digoxin induced arrhythmia | 60 min | 4 mg/kg/min  IV infusion | ↓ Premature atrial contractions, Ventricular extrasystole, VT and VF |  | (13) |
| *Cynodon dactylon* (L.) Pers. | hydroalcoholic extract of rhizomes | Male SD rats isolated heart/  I/R-induced arrhythmia | 60 min | 25, 50, 100 and 200 μg/ml  Perfusion | ↓ in the number and duration of VT  ↓ total number of ischemic VEBs  ↓ total VF incidences  ↓ reversible VF duration | Antioxidant  Anti-inflammatory | (14) |
| *Dracocephalum moldavica*L*.* | Methanolic extract of Aerial parts | Male SD rats isolated heart/  I/R-induced arrhythmia | 150 min | 25, 50 and 200 μg/ml  Perfusion | ↓ incidence and duration of VT  ↓ reversible VF duration  ↓ the number of VT and total VEBs | Antioxidant  Inhibition of Ca channels  ↑ release NO | (15) |
| *Ficus carica*L. | Methanolic extract of leaves | Male Wistar rats isolated heart/  I/R-induced arrhythmia | 140 min | 0.04, 0.2 and 1 mg/ml  Perfusion | ↓ number of Single, Salvos, and total VEBs, especially by higher concentrations  ↓ incidence and duration of reversible VF | Antioxidant | (16) |
| *Ginkgo biloba*L. | EGb761 (a standardized extract from leaves) | Male Wistar rats/ I/R-induced arrhythmia | 40 min | 25,50,100 and 200 mg/kg  IP injection | dose-dependent inhibitory effects on the incidence of VA | Antioxidant activity;  Other potential mechanisms: (bilobalide) inhibition of HCN channel-mediated pacemaker current (17) | (18) |
|  | EGb761 | Male SD rats isolated heart/  I/R-induced arrhythmia | 10 days | 25 and 50 mg/kg/day  PO | ↓ incidence of re-perfusion-induced VF  ↓ incidence of re-perfusion-induced VT |  | (19) |
|  | EGb71 | Male SD rats isolated heart/  I/R-induced arrhythmia | 10 days | 25,50, 100 or 200 mg/kg/day  PO | ↓ the incidence of reperfusion-induced VF and VT |  | (20) |
|  |  |  | Acute administration | 50 and 200 mg/l  Perfusion | ↓ incidence of reperfusion-induced arrhythmias |  |  |
| *Vitis vinifera*L. | Grape pomace | Male Wistar rats/  I/R-induced arrhythmia | 6 weeks | 1g/kg/day  PO | ↓ incidence and severity of reperfusion-induced arrhythmias | VII | (21) |
| *Gynostemma pentaphyllum*(Thunb.) Makino | Aques extract of leaves | Male guinea-pigs/ pitressin or ouabain induced arrhythmia | Acute administration | 2.5, 5 and 10 mg/kg  IV injection | ↓ incidence of ventricular arrhythmias.  Reversion of the persistent VT back to normal and stable sinus rhythm in a dose-dependent manner. | Inhibition of Ca channels | (22) |
| *Harpagophytum procumbens*(Burch.) DC. ex Meisn | Dried methanolic extract of root | Male SD rats/  Aconitine, epinephrine, chloroform or CaCl_2_-induced arrhythmia | Acute administration | 100,200,300 and 400 mg/kg  PO | ↓ susceptibility to cardiac arrhythmias induced by aconitine, CaCl_2 ._  Preventing from ventricular extrasystoles and auricular block.  An antiarrhythmic effect equal to or higher than lidocaine. | Inhibition of Ca channels | (23) |
|  |  | rabbit isolated heart/  Aconitine, epinephrine, chloroform or CaCl_2_-induced arrhythmia | Acute administration | 1 mg/ 0.25 ml/ 15s  Perfusion |  |  |  |
|  |  | Rat isolated heart/  I/R-induced arrhythmia | Acute administration | 1 and 2 mg  Perfusion | ↓ Hyperkineric ventricular arrhythmia |  | (24) |
| *Marrubium crassidens*Boiss. | Methanol extract of aerial parts | Male Wistar rat isolated heart/  I/R-induced arrhythmia | 120min | 10, 50 and 100 μg/ml  Perfusion | ↓ number of VT with 10 and 100 μg/ml  ↓ number of single, triplet and total VEBs with 100 μg/ml of extract | Antioxidant  Inhibition of Ca channels | (25) |
| *Melissa officinalis*L. | Ethanolic extract of *dried leaves* | Male SD rat/  I/R-induced arrhythmia | 14 days | 50, 100 or 200 mg/kg/day  PO | ↓ mean total number of VEB episodes  ↓ mean total number of VT episodes  ↓ duration of VTs | Antioxidant | (26) |
|  | Aques extract of the dried aerial  parts | Male Wistar rats /  I/R-induced arrhythmia | Acute administration | 50, 100, 200 and 400 mg/ml/kg  IP injection | ↓ number of VFs  ↓ score of arrhythmia severity |  | (27) |
| *Mentha × villosa*Huds. | Aqueous extract of leaves | MaleSD rats isolated heart/  I/R-induced arrhythmia | 10 min | 3.6 mg/l  Perfusion | ↓ incidence and duration of VT and VF | Antioxidant | (28) |
| *Panax ginseng*C.A.Mey. | RSE (a standardized root extract) | Male rats /  I/R-induced arrhythmia | 60 min before experiment | 20, 40, 80 mg/kg  PO | ↓ incidence of BG, TG,VT, and VF with RSE 80mg/kg dosing | ↑ release NO | (29) |
| *Potentilla reptans*L. | Dried root ethyl acetate fraction | Wistar rats isolated heart /  I/R-induced arrhythmia | 40 min before experiment | 1, 2 and 3 µg/ml  Perfusion | ↓ incidence of VF  ↓ arrhythmia severity score | Antioxidant  ↑ release NO | (30) |
| *Rhodiola rosea*L. | Water extract of *Rhodiola Rosea* | Wistar rats/  I/R-induced arrhythmia | 5 days | 16 mg/kg  Intragastric | ↓ the incidence of VT, ventricular extrasystole  ↓ Arrhythmia severity index | ↑ Opioid peptides in the blood and tissues of rats | (1) |
| *Rosmarinus officinalis* L. | hydroalcoholic extract of rosemary leaves | Male SD rats/  CaCl_2_-induced arhhythmia | 14 days | 50,100 and 200 mg/kg/day  PO | ↓ incidence of PVB  ↓ rate of VT and VF in the groups receiving 100 and 200 mg/kg of the extract | ↓Ca ions availability in the cardiomyocytes | (31) |
| *Scrophularia frigida*Boiss. | methanolic extract of aerial parts | Wistar rat isolated heart /  I/R-induced arrhythmia | 150 min | 1, 5, and 10 μg/ml  Perfusion | ↓ the number of single and total arrhythmias  ↓ number and duration of VT | Inhibition of fibrosis and remodeling (32) | (33) |
| *Sophora flavescens*Aiton | Ethanolic extract of dried root | male SD rats/  I/R-induced arrhythmia | Acute administration | 60 and 120 mg/kg  IV injection | ↓ incidence of VT and VF  ↑ onset time of VT and VF | Modulation of K & Ca currents | (34) |
|  |  | male ICR mice/  Aconitin induced arhythmia | Acute administration | 120, 180 and 240 mg/kg | ↑ onset time of VT and VF |  |  |
|  | Ethanolic extract of dried root | mongrel dogs/  Electrical pulse induced arrhythmia | Acute administration | 120 mg/kg  IV injection | ↑VF threshold |  | (35) |
| *Tinospora cordifolia*(Willd.) Miers | Alcoholic extract of whole plant | Male Wistar rats/  CaCl_2_-induced arhhythmia | Acute administration | 150, 250, 450 mg/kg  IV injection | ↓ atrial and ventricular fibrillation | Normalizing the overload of calcium | (36) |
| *Zingiber officinale*Roscoe | White ginger powder | Male Wistar rats/  CaCl_2_-induced arhhythmia | 15 days | 100 mg/kg/day  PO | ↓ incidence of VT and VF  ↓ incidence of VPB | Antioxidant | (37) |

Supplementary Table 2: Multi-component herbal preparations with antiarrhythmic effects and their probable mechanism of action

| Multi-component herbal preparations | | | | | | |
| --- | --- | --- | --- | --- | --- | --- |
| Formulation name | Study design | duration | Dosage/  intervention | Outcomes | Probable Mechanisms | References |
| Catuama preparation  (*Trichilia catigua, Paullinia cupana, Ptychopetalum olacoides, and Zinziber officinalis*) | Rabbits isolated heart/  electrical burst-induced VF | 20 min | 200 μg/mL  Perfusion | Reversion of VF  Complete protection on arrhythmia induction | Inhibition of Ca channels | (38) |
| Dingxin Recipe  (the seeds of  *Ziziphus* *jujuba* var. *spinosa* (Bunge) Hu ex H.F.Chow, the root of *Sophora* *flavescens* Aiton, the root of *Coptis* *chinensis* Franch,  *Wolfiporia extensa,* the root of *Codonopsis pilosula (Franch.) Nannf,* the root of *Salvia* *miltiorrhiza* Bunge, the root of *Paeonia* *lactiflora* Pall, the dried fruit of *Trichosanthes* *kirilowii* Maxim, Ganoderma Lucidum Seu Japonicum, dried root of *Panax* *notoginseng* (Burkill) F.H.Chen | Male Wistar rats/  I/R-induced arrhythmia | 7 days | 12.5 g/kg/day  intragastric | ↓incidence of VT & VF  ↓ mean duration of VT & VF  ↓ Arrhythmia severity scores | VII  Other potential mechanisms:  V (39) | (40) |

Supplementary Table 3: phytochemicals with antiarrhythmic effects and their probable mechanism of action

| Phytochemicals | | | | | | |
| --- | --- | --- | --- | --- | --- | --- |
| Phytochemical | Study design | Duration | Dosing/  Intervention | outcomes | Probable Mechanisms | references |
| Alkaloids | | | | | | |
| Dauricine | Mongrel dogs/  I/R-induced arrhythmia | 30 min | 5mg/kg IV bolous followed by  0.1mg/kg  IV infusion | ↓ incidence VEB, VT and VF | Inhibition of K & Ca channels (41, 42) | (43) |
| Guanfu total base  (Alkaloid extract of *Aconitum coreanum*) | Male SD rats/  Ach-CaCl_2_ induced arrhythmia | 4 days | 45mg/kg/day  PO | ↓AF duration | Inhibition of apoptosis  Inhibition of connexins(44) | (45) |
| Guanfu base A | Rats/  Aconitine-induced arrhythmia | Acute administration | 14, 7, and 3.5 mg/kg  IV injection | Suppression of VT and ventricular ectopia | Selective inhibition of late sodium current  And inhibition of hERG channels (46) | (47) |
| Neferine | Mongrel dogs/  I/R-induced arrhythmia  And Programmed Electrical Stimulation | Acute administration | 8mg/kg  IV injection | ↓ incidence VT and VF | inhibition of hERG channels  (48) | (49) |
| Flavonoids | | | | | | |
| TFCF or TFC | Wistar rats/  aconitine-induced arrhythmia | 7 days | 0.1, 0.2, or 0.4g/kg/day of TFCF or 0.2 g/kg/day of TFC  PO | ↓ susceptibility to VE, VT, and VF | inhibition of Na channels | (50) |
| Dioclein | Male Wistar rats isolated herat/  I/R-induced arrhythmia | 15 min | 30, 150 and 300 nM  Perfusion | ↓ incidence of arrhythmia  ↓ arrhythmia severity score | Antioxidant | (51) |
| Grape seed oligomeric Procyanidins extracts | Male SD rats isolated heart /  I/R-induced arrhythmia | 21 days | 3g/100g/day  PO | ↓ incidence of VF  ↓ duration of VF  ↑ mean duration of normal sinus rhythm | Antioxidant | (52) |
| Flavonoid  fraction of *Plantago major* | Male myocardium/  Electrical stimulation-induced arrhythmia | Acute administration | 0.01 – 0.05 mg/mL  Perfusion | ↓ incidence of arrhythmia and tachycardia | inhibition of Na and Ca channels | (53) |
| Furanocoumarin | | | | | | |
| Bergamottin | Male ginea pig/ Pitressin or ouabain-induced arrhythmias | Acute administration | 0.25, 0.5 and 1 mg.kg  IV injection | ↓ incidence of cardiac arrhythmias  ↓ susceptibility to VEB and VT  Reversion of persistent VT | Inhibition of Ca channels | (54) |
|  | Male SD rats isolated heart/  I/R-induced arrhythmia | 30 min | 10^-6^, 10^-5^ and 10^-4^ g/mL  Perfusion | ↓ incidence of VT, VF and VPB  ↓ severity of arrhythmias |  |  |
| Lignin | | | | | | |
| Honokiol | Male SD rats/  I/R-induced arrhythmia | Acute administration | 10^-7^, 10^-8^, and 10^-9^ g/kg  IV injection | ↓ incidence of VT and VF  ↓ Duration of VT and VF | Antioxidant | (55) |
| Liriodendrin | Male SD rats/  CaCl_2_-induced arrhythmia | Acute administration | 5 mg/kg  IV injection | onset time of arrhythmia  ↓incidence of VF | Anti-inflammatory (56) | (57) |
| Phenolic acids | | | | | | |
| Bergenin | Male SD rats/  I/R-induced arrhythmia or  BaCl_2_-induced arrhythmia | Acute administration | 0.2, 0.4 and 0.8 mg/kg  IV injection | ↓ duration of VPB, VT and VF | Antioxidant | (58) |
|  | Rabbit/  Electrical stimulation-induced AF | Acute administration | 0.8mg/kg  IV injection | ↑AF thereshold |  |  |
| Rosmarinic acid | Male SD rats isolated heart/  I/R-induced arrhythmia | 10 min | 1×10^-5^ mol/L  Perfusion | ↓incidence of VPB, VF | Antioxidant (59) | (28) |
| Gallic acid | Male SD rats/  CaCl_2_-induced arrhythmia | 10 days | 10, 30 and 50 mg/kg/day  gavage | ↓ incidence of VPB, VT and VF  More effective than quinidine. | Inhibition of platelet aggregation  Antioxidant | (60) |
| Ellagic acid | Male SD rats/  CaCl_2_-induced arrhythmia | 10 days | 15 mg/kg/day  gavage | ↓ incidence of VT, VF and VPB | HMG coA reductase inhibitor  Antioxidant | (61) |
| Terpenoids | | | | | | |
| Crocin | Male Wistar rats/ Male SD rats/ | 3 weeks | 20 mg/kg  IP injection | ↓ incidence of VEB, VF and VT  ↓ duration of VT | Antioxidant | (62) |
| Ginkgolide B | Male SD rats isolated heart/ Male SD rats/ | 70min | 1.5, 3, 6 ×10 ^-5^ and 1.2 × 10 ^-4^ mol/1  perfusion | ↓ incidence of VT, VF and PVB | Inhibition of potassium and calcium channels (17) | (63) |
| Thymoquinone | Male Wistar rats Isolated atria/ Ouabain-induced arrhythmia | 30 min | 10, 20 and 40 µM  incubation | ↑ the onset time of arrhythmia  ↓ the intensity of arrhythmia | Activation of muscarinic and serotonergic systems in heart | (64) |
| 3,4-seco-Lupane triterpenes  (Chiisanoside, divaroside, sessiloside-A1, and chiisanogenin) | Wistar rat/  BaCl_2_- induced arrhythmia | Acute administration | (Chiisanoside:  50 mg/kg  Divaroside:  41.6 mg/kg  Sessiloside-A1: 32.5 mg/kg Chiisanogenin:  25mg/kg)  IP injection | all the four 3,4-seco-lupane triterpenes caused a significant reduction in duration of VA, PVC and VT | Antioxidant | (65) |
| Lipid | | | | | | |
| Trilinolein | Male SD rats/  I/R-induced arrhythmia | Acute administration | 10^-11^, 10^-10^, 10^-9^, 10^-8^ and 10^-7^ g/kg  IV injection | ↓ total number of ectopic beats  ↓incidence of VT  ↓ durationof VT and VF | NI | (66) |

**References**

1. Maslov LN, Lishmanov YB, Arbuzov AG, Krylatov AV, Budankova EV, Konkovskaya YN, et al. Antiarrhythmic activity of phytoadaptogens in short-term ischemia-reperfusion of the heart and postinfarction cardiosclerosis. Bulletin of experimental biology and medicine. 2009;147(3):331-4.

2. Martín N, Bardisa L, Pantoja C, Vargas M, Quezada P, Valenzuela J. Anti-arrhythmic profile of a garlic dialysate assayed in dogs and isolated atrial preparations. Journal of ethnopharmacology. 1994;43(1):1-8.

3. Sungnoon R, Kanlop N, Chattipakorn SC, Tawan R, Chattipakorn N. Effects of garlic on the induction of ventricular fibrillation. Nutrition (Burbank, Los Angeles County, Calif). 2008;24(7-8):711-6.

4. Fattahi M, Dalir-Naghadeh B, Maham M. Prophylactic and therapeutic effects of garlic extract on Nerium oleander-induced arrhythmia: a new approach to antiarrhythmic therapy in an ovine model. Clinical toxicology (Philadelphia, Pa). 2013;51(8):737-47.

5. Rietz B, Isensee H, Strobach H, Makdessi S, Jacob R. Cardioprotective actions of wild garlic (allium ursinum) in ischemia and reperfusion. Molecular and cellular biochemistry. 1993;119(1-2):143-50.

6. Sharma RK, Sharma AK, Mohan G. Evaluation of cardioprotective activity of aqueous and ethanolic extract of bauhinia variegata in cacl2 induced arrhythmia in albino rats. Journal of Applied Pharmaceutical Science. 2013;3(7):169.

7. Sedighi M, Nazari A, Faghihi M, Rafieian-Kopaei M, Karimi A, Moghimian M, et al. Protective effects of cinnamon bark extract against ischemia–reperfusion injury and arrhythmias in rat. Phytotherapy Research. 2018;32(10):1983-91.

8. Al-Yahya MA, Mothana RA, Al-Said MS, El-Tahir KE, Al-Sohaibani M, Rafatullah S. Citrus medica "Otroj": attenuates oxidative stress and cardiac dysrhythmia in isoproterenol-induced cardiomyopathy in rats. Nutrients. 2013;5(11):4269-83.

9. Joukar S, Ghasemipour-Afshar E, Sheibani M, Naghsh N, Bashiri A. Protective effects of saffron (Crocus sativus) against lethal ventricular arrhythmias induced by heart reperfusion in rat: a potential anti-arrhythmic agent. Pharmaceutical biology. 2013;51(7):836-43.

10. Veveris M, Koch E, Chatterjee SS. Crataegus special extract WS® 1442 improves cardiac function and reduces infarct size in a rat model of prolonged coronary ischemia and reperfusion. Life Sciences. 2004;74(15):1945-55.

11. Garjani A, Nazemiyeh H, Maleki N, Valizadeh H. Effects of extracts from flowering tops of Crataegus meyeri A. Pojark. on ischaemic arrhythmias in anaesthetized rats. Phytotherapy research : PTR. 2000;14(6):428-31.

12. al Makdessi S, Sweidan H, Dietz K, Jacob R. Protective effect of Crataegus oxyacantha against reperfusion arrhythmias after global no-flow ischemia in the rat heart. Basic research in cardiology. 1999;94(2):71-7.

13. Alp H, Soner BC, Baysal T, Şahin AS. Protective effects of Hawthorn (Crataegus oxyacantha) extract against digoxin-induced arrhythmias in rats. Anatolian journal of cardiology. 2015;15(12):970-5.

14. Najafi M, Nazemiyeh H, Ghavimi H, Gharakhani A, Garjani A. Effects of hydroalcoholic extract of Cynodon dactylon (L.) pers. on ischemia/reperfusion-induced arrhythmias. Daru. 2008;16(4):233-8.

15. Najafi M, Ghasemian E, Fathiazad F, Garjani A. Effects of total extract of Dracocephalum moldavica on ischemia/reperfusion induced arrhythmias and infarct size in the isolated rat heart. Iranian Journal of Basic Medical Sciences. 2009;11(4):229-35.

16. Allahyari S, Delazar A, Najafi M. Evaluation of general toxicity, anti-oxidant activity and effects of Ficus carica leaves extract on ischemia/reperfusion injuries in isolated heart of rat. Advanced pharmaceutical bulletin. 2014;4(Suppl 2):577.

17. Zhao X, Yao H, Yin H-L, Zhu Q-L, Sun J-L, Ma W, et al. Ginkgo biloba extract and ginkgolide antiarrhythmic potential by targeting hERG and ICa-L channel. Journal of pharmacological sciences. 2013:13118FP.

18. Shen J, Wang J, Zhao B, Hou J, Gao T, Xin W. Effects of EGb 761 on nitric oxide and oxygen free radicals, myocardial damage and arrhythmia in ischemia-reperfusion injury in vivo. Biochimica et biophysica acta. 1998;1406(3):228-36.

19. Tosaki A, Pali T, Droy-Lefaix MT. Effects of Ginkgo biloba extract and preconditioning on the diabetic rat myocardium. Diabetologia. 1996;39(11):1255-62.

20. Tosaki A, Droy-Lefaix MT, Pali T, Das DK. Effects of SOD, catalase, and a novel antiarrhythmic drug, EGB 761, on reperfusion-induced arrhythmias in isolated rat hearts. Free radical biology & medicine. 1993;14(4):361-70.

21. Perdicaro DJ, Lanzi CR, Fontana AR, Antoniolli A, Piccoli P, Miatello RM, et al. Grape pomace reduced reperfusion arrhythmias in rats with a high-fat-fructose diet. Food & function. 2017;8(10):3501-9.

22. Circosta C, De Pasquale R, Occhiuto F. Cardiovascular effects of the aqueous extract of Gynostemma pentaphyllum Makino. Phytomedicine : international journal of phytotherapy and phytopharmacology. 2005;12(9):638-43.

23. Circosta C, Occhiuto F, Ragusa S, Trovato A, Tumino G, Briguglio F, et al. A drug used in traditional medicine: Harpagophytum procumbens DC. II. Cardiovascular activity. Journal of ethnopharmacology. 1984;11(3):259-74.

24. Costa De Pasquale R, Busa G, Circosta C, Iauk L, Ragusa S, Ficarra P, et al. A drug used in traditional medicine: Harpagophytum procumbens DC. III. Effects on hyperkinetic ventricular arrhythmias by reperfusion. Journal of ethnopharmacology. 1985;13(2):193-9.

25. Rameshrad M, Vaez H, Toutounchi NS, Fathiazad F, Garjani A. Effect of methanolic extract of Marrubium crassidens Boiss on Ischemia/Reperfusion induced arrhythmias and infarct size in isolated rat heart. Pharmaceutical Sciences. 2014;20(3):80-9.

26. Sedighi M, Faghihi M, Rafieian-Kopaei M, Rasoulian B, Nazari A. Cardioprotective effect of ethanolic leaf extract of Melissa officinalis L against regional ischemia-induced arrhythmia and heart injury after five days of reperfusion in rats. Iranian Journal of Pharmaceutical Research. 2019;18(3):1530-42.

27. Joukar S, Zarisfi Z, Sepehri G, Bashiri A. Efficacy of melissa officinalis in suppressing ventricular arrhythmias following ischemia-reperfusion of the heart: A comparison with amiodarone. Medical Principles and Practice. 2013;23(4):340-5.

28. Brosková Z, Drábiková K, Sotníková R, Fialová S, Knezl V. Effect of plant polyphenols on ischemia-reperfusion injury of the isolated rat heart and vessels. Phytotherapy Research. 2013;27(7):1018-22.

29. Zhou H, Hou SZ, Luo P, Zeng B, Wang JR, Wong YF, et al. Ginseng protects rodent hearts from acute myocardial ischemia-reperfusion injury through GR/ER-activated RISK pathway in an endothelial NOS-dependent mechanism. Journal of ethnopharmacology. 2011;135(2):287-98.

30. Enayati A, Yassa N, Mazaheri Z, Rajaei M, Pourabouk M, Ghorghanlu S, et al. Cardioprotective and anti-apoptotic effects of Potentilla reptans L. root via Nrf2 pathway in an isolated rat heart ischemia/reperfusion model. Life sciences. 2018;215:216-26.

31. Shoja N, Dianat M, Hoseyni Nik SM, Ramazani G. The evaluation of the protective effects of the hydro-alcoholic extract of rosemary (Rosmarinus officinalis L.) on ventricular arrhythmias in rats. Journal of Babol University of Medical Sciences. 2015;17(5):66-72.

32. Huang XY, Chen CX, Zhang XM, Liu Y, Wu XM, Li YM. Effects of ethanolic extract from Radix Scrophulariae on ventricular remodeling in rats. Phytomedicine : international journal of phytotherapy and phytopharmacology. 2012;19(3-4):193-205.

33. Garjani A, Vaez H, Delazar A, Rameshrad M, Afshar FH, Asgharian P. Cardioprotective effects of methanolic extract of Scrophularia frigida on ischemia-reperfusion-induced injuries in isolated rat heart. Iranian Journal of Pharmaceutical Research. 2017;16:35-45.

34. Dai S, Chan MY, Lee SS, Ogle CW. The antiarrhythmic effects of Sophora flavescens Ait. in rats and mice. The American journal of Chinese medicine. 1986;14(3-4):119-23.

35. Dai S, Chan M-Y, Lee S-S, Pharm B, Ogle CW. Effects of Sophora flavescens Ait: on Haemodynamics and Ventricular Fibrillation Threshold in Anaesthized Dogs. The American journal of Chinese medicine. 1987;15(01n02):53-7.

36. Sharma AK, Kishore K, Sharma D, Srinivasan BP, Agarwal SS, Sharma A, et al. Cardioprotective activity of alcoholic extract of Tinospora cordifolia (Willd.) Miers in calcium chloride-induced cardiac arrhythmia in rats. Journal of Biomedical Research. 2011;25(4):280-6.

37. Karbalaei N, Hamzavi GR. Negative chronotropic and antiarrhythmic effects of ginger (Zingiber officinale) and intermittent fasting on Cacl2 induced-arrhythmia. Research Journal of Pharmaceutical, Biological and Chemical Sciences. 2016;7(1):978-83.

38. Pontieri V, Neto AS, de França Camargo AF, Koike MK, Velasco IT. The herbal drug Catuama reverts and prevents ventricular fibrillation in the isolated rabbit heart. Journal of electrocardiology. 2007;40(6):534.e1-8.

39. Chen R-c, Sun G-b, Ye J-x, Wang J, Sun X-b. Salvianolic acid B attenuates doxorubicin-induced ER stress by inhibiting TRPC3 and TRPC6 mediated Ca2+ overload in rat cardiomyocytes. Toxicology Letters. 2017;276:21-30.

40. Jia YH, Zhang YX, Li LJ, Liu YW, Li CH, Fu XQ, et al. Dingxin Recipe Prevents Ischemia/ReperfusionInduced Arrhythmias via Up-regulating Prohibitin and Suppressing Inflammatory Responses. Chinese journal of integrative medicine. 2012;18(2):120-9.

41. Xia J-S, Guo D-L, Zhang Y, Zhou Z-N, Zeng F-D, Hu C-J. Inhibitory effects of dauricine on potassium currents in guinea pig ventricular myocytes. Acta Pharmacologica Sinica. 2000;21(1):60-4.

42. Guo D, Zhou Z, Zeng F, Hu C. Dauricine inhibited L-type calcium current in single cardiomyocyte of guinea pig. Zhongguo yao li xue bao= Acta Pharmacologica Sinica. 1997;18(5):419-21.

43. Zhu J, Zeng F, Hu C. Protective and anti-arrhythmic effects of dauricine and verapamil on acute myocardial infarction in anesthetized dogs. Zhongguo yao li xue bao= Acta Pharmacologica Sinica. 1992;13(3):249-51.

44. Tang Y, Yin Y, Huang L, Wang M, Yu X, Xu J. Effects of Guanfu total base on experimental atrial fibrillation. Guide Chin Med. 2011;9:249-51.

45. Li Y, Song B, Xu C. Effects of Guanfu total base on Bcl-2 and Bax expression and correlation with atrial fibrillation. Hellenic Journal of Cardiology. 2018;59(5):274-8.

46. Si-Si J, Qiao G, Jing X, Peng Y, Jing-Han L, Yi-Qun T. Antiarrhythmic ionic mechanism of Guanfu base A—Selective inhibition of late sodium current in isolated ventricular myocytes from guinea pigs. Chinese journal of natural medicines. 2015;13(5):361-7.

47. Xiong F, Liu K, Liu S, Chen J, Liu J, Wang H, et al. Safety, heart specificity, and therapeutic effect evaluation of Guanfu base A-loaded solid nanolipids in treating arrhythmia. Drug Delivery and Translational Research. 2018;8(5):1471-82.

48. Gu DF, Li XL, Qi ZP, Shi SS, Hu MQ, Liu DM, et al. Blockade of HERG K+ channel by isoquinoline alkaloid neferine in the stable transfected HEK293 cells. Naunyn-Schmiedeberg's Archives of Pharmacology. 2009;380(2):143-51.

49. Zhibin G, Qing L, Hongyu C, Zhi X. Antiarrhythmic efficacy of neferine assessed by programmed electrical stimulation in a canine model of electropharmacology. Journal of Chinese Pharmaceutical Sciences. 2002;11(2):35-42.

50. Qiu M, Dong YH, Han F, Qin JM, Zhang HN, Du JX, et al. Influence of total flavonoids derived from Choerospondias axillaris folium on aconitine-induced antiarrhythmic action and hemodynamics in Wistar rats. Journal of toxicology and environmental health Part A. 2016;79(19):878-83.

51. Vianna HR, Cortes SF, Ferreira AJ, Capettini LS, Schmitt M, Almeida AP, et al. Antiarrhythmogenic and antioxidant effect of the flavonoid dioclein in a model of cardiac ischemia/reperfusion. Planta medica. 2006;72(4):300-3.

52. Makdessi SA, Sweidan H, Jacob R. Effect of oligomer procyanidins on reperfusion arrhythmias and lactate dehydrogenase release in the isolated rat heart. Arzneimittel-Forschung/Drug Research. 2006;56(5):317-21.

53. Khushmatov SS, Makhmudov RR. Antiarrhythmic Activity of the Flavonoid Fraction of Plantago Major L. Extract. Pharmaceutical Chemistry Journal. 2019;52(12):992-5.

54. Occhiuto F, Circosta C. Antianginal and antiarrhythmic effects of bergamottine, a furocoumarin isolated from bergamot oil. Phytotherapy Research. 1996;10(6):491-6.

55. Tsai SK, Huang SS, Hong CY. Myocardial protective effect of honokiol: an active component in Magnolia officinalis. Planta medica. 1996;62(6):503-6.

56. Jung H-J, Park H-J, Kim R-G, Shin K-M, Ha J, Choi J-W, et al. In vivo anti-inflammatory and antinociceptive effects of liriodendrin isolated from the stem bark of Acanthopanax senticosus. Planta medica. 2003;69(07):610-6.

57. Feng C, Li BG, Gao XP, Qi HY, Zhang GL. A new triterpene and an antiarrhythmic liriodendrin from Pittosporum brevicalyx. Archives of pharmacal research. 2010;33(12):1927-32.

58. Pu H-L, Huang X, Zhao J-H, Hong A. Bergenin is the antiarrhythmic principle of Fluggea virosa. Planta medica. 2002;68(04):372-4.

59. Han J, Wang D, Ye L, Li P, Hao W, Chen X, et al. Rosmarinic acid protects against inflammation and cardiomyocyte apoptosis during myocardial ischemia/reperfusion injury by activating peroxisome proliferator-activated receptor gamma. Frontiers in pharmacology. 2017;8:456.

60. Dianat M, Akbari GH, Badavi M. Antidysrhythmic Effects of Gallic acid on cacl2-induced arrhythmia in rat. International Journal of Research and Development in Pharmacy & Life Sciences. 2013;2(6):686-9.

61. Dianat M, Amini N, Badavi M, Farbood Y. Ellagic acid improved arrhythmias induced by CaCL2 in the rat stress model. Avicenna Journal of Phytomedicine. 2015;5(2):120.

62. Jahanbakhsh Z, Rasoulian B, Jafari M, Shekarforoush S, Esmailidehaj M, taghi Mohammadi M, et al. Protective effect of crocin against reperfusion-induced cardiac arrhythmias in anaesthetized rats. EXCLI journal. 2012;11:20.

63. Koltai M, Tosaki A, Hosford D, Braquet P. Ginkgolide B protects isolated hearts against arrhythmias induced by ischemia but not reperfusion. European journal of pharmacology. 1989;164(2):293-302.

64. Malihi G, Nikoui V, Pousti A, Azam B. Cardiac benefits of black seed extract: Its main compound thymoquinone inhibits ouabain-induced arrhythmia in isolated rat atria. International Journal of Pharmaceutical Research. 2017;9(1):66-73.

65. Zhao Y, Wang X, Chen C, Shi K, Li J, Du R. Protective Effects of 3,4-Seco-Lupane Triterpenes from Food Raw Materials of the Leaves of Eleutherococcus Senticosus and Eleutherococcus Sessiliflorus on Arrhythmia Induced by Barium Chloride. Chem Biodivers. 2021;18(4):e2001021.

66. Chan P, Tsai SK, Chiang BN, Hong CY. Trilinolein reduces infarct size and suppresses ventricular arrhythmias in rats subjected to coronary ligation. Pharmacology. 1995;51(2):118-26.
